# Species delimitation, discovery and conservation in a tiger beetle species complex despite discordant genetic data

**DOI:** 10.1038/s41598-024-56875-9

**Published:** 2024-03-19

**Authors:** Daniel P. Duran, Robert A. Laroche, Stephen J. Roman, William Godwin, David P. Herrmann, Ethan Bull, Scott P. Egan

**Affiliations:** 1 Department of Environmental Science, Rowan University, Glassboro, NJ 08028 USA; 2 Department of BioSciences, Rice University, Houston, TX 77005 USA; 3 Florida State Collection of Arthropods, Gainesville, FL 32608 USA; 4 Sam Houston State Natural History Collection, Huntsville, TX 77340 USA; 5 Alyn Patrick and Associates, Irving, TX 75063 USA

**Keywords:** Ecology, Evolution

## Abstract

In an age of species declines, delineating and discovering biodiversity is critical for both taxonomic accuracy and conservation. In recent years, there has been a movement away from using exclusively morphological characters to delineate and describe taxa and an increase in the use of molecular markers to describe diversity or through integrative taxonomy, which employs traditional morphological characters, as well as genetic or other data. Tiger beetles are charismatic, of conservation concern, and much work has been done on the morphological delineation of species and subspecies, but few of these taxa have been tested with genetic analyses. In this study, we tested morphologically based taxonomic hypotheses of polymorphic tiger beetles in the *Eunota circumpicta* (LaFerté-Sénectère, 1841) species complex using multilocus genomic and mtDNA analyses. We find multiple cryptic species within the previous taxonomic concept of *Eunota circumpicta*, some of which were historically recognized as subspecies. We found that the mtDNA and genomic datasets did not identify the same taxonomic units and that the mtDNA was most at odds with all other genetic and morphological patterns. Overall, we describe new cryptic diversity, which raises important conservation concerns, and provide a working example for testing species and subspecies validity despite discordant data.

## Introduction

The discovery and description of new biodiversity during an era of rapid species declines is vital for all the life sciences, especially because biodiversity is critical for the health of ecosystems and the human societies that rely on them worldwide^1–4^. Without knowing the proper taxonomic or evolutionary units present in a given region, scientists and public policy makers cannot start the process of knowing what to conserve. This is a serious challenge, given our knowledge of the importance of biodiversity, from supporting healthy ecosystems, which produce ecosystem services for humans, to being an essential component in the solution to climate change^5^.

Taxonomy is the description of biodiversity and its classification into groups; groups which putatively reflect distinct evolutionary lineages^6^. Taxonomic accuracy is essential for effective conservation efforts^7^. The unit of biodiversity which has received the most attention in taxonomy and conservation is species^8,9^, and some have argued that this is the most “real” unit of taxonomy, with higher taxa representing historical entities^10–12^. In many groups of organisms, the subspecies has become a key taxonomic unit for conservation efforts^13–16^, even though this taxonomic concept is much less clearly defined and has been the subject of considerable controversy^17–19^. Given that the species and subspecies levels of taxonomy are most often the focal point of conservation work, the establishment of taxonomy that appropriately represents the divergence between these units is a critical prerequisite to any conservation assessment^20^. In some cases, taxonomic confusion has led to species being mistakenly included or excluded from conservation efforts, or even erroneously considered threatened or extinct, thus, diverting limited resources away from other threatened taxa^21–23^.

Species delimitation has been traditionally based on morphological characters for the vast majority of eukaryotic taxa, with a lesser reliance on behavioral, ecological or other characters^24^. This model is implicitly based on the idea that fixed morphological differences in two or more sets of populations are the result of the splitting of gene pools from a single ancestral taxon. This method of recognizing species as entities that are consistently distinct with respect to body structures is termed the morphological species concept^25^ and its derivatives^26^. In recent decades, taxonomists have incorporated molecular data into taxonomic revisions of species-groups and, to a lesser degree, species descriptions^27^, resulting in significant changes to established taxonomic frameworks that are incongruous with the MSC^28–32^. Starting in the early 2000s there has been a trend towards a heavy reliance on purely molecular data, including the use of mitochondrial DNA (mtDNA) for ‘DNA Barcoding’^33^. This sea change presented challenges for the taxonomic community on how to best reconcile and incorporate these multiple types of data^34^.

The idea that all species are lineages, and that multiple lines of evidence may be used to identify them has been termed the general lineage species concept^35,36^. If different sets of data, molecular, morphological, or other, are to be used, how best to integrate them? For years, cladistic systematists have argued for a more synthetic “total evidence” approach based on the Popperian philosophy that all available data should be used when making systematic inferences^37,38^. The approach is often to incorporate independent datasets into a single concatenated analysis^39^. Another method is to employ a ‘taxonomic congruence’ approach, where multiple datasets are separately analyzed, and taxonomic hypotheses are evaluated based on the consensus of all datasets^40–42^. Often, there is broad agreement between data types, including different molecular markers based on either mtDNA sequences or genomic markers, such as multilocus SNP data. Many species delineation efforts have utilized the complementary signals in different genetic, morphological and/or geographic datasets^31,42^. However, in some cases, there may be conflicting signals, with different patterns observed in mitochondrial and genomic data, known as mitonuclear discordance. Resolving taxonomic relationships in the face of genetic discordance is an important challenge for biodiversity science^43^.

Tiger beetles are one of the most charismatic and popular groups of insects worldwide^44^ and a global ‘flagship’ taxon for insect conservation^45^. Renowned for using their highly developed vision and extreme speed when hunting prey, tiger beetles occupy nearly all terrestrial ecosystems and are distributed across the globe^46,47^. Many species are iridescent, metallic, and/or highly variable in color. The approximately 3000 described species worldwide^48^ are mostly concentrated in the tropical parts of the world, however; species of tiger beetles can be found on all continents except Antarctica. They thrive in dynamic and primarily open habitats such as sea beaches, sand dunes, alpine meadows, riverbanks, clay banks, tidal and alkali flats, rock outcroppings, trails, and openings in forests. Despite the public appeal and scientific interest of this clade, the morphology-driven species-level taxonomy of the group is critically out of date. Most species were described in the nineteenth century based on morphology, but recent research on North American species reveals rampant incongruity between morphologically defined groups and other data sources, including genetic, genomic, behavioral and ecological data^32,42,49,50^. Generally, these studies find that (1) species with large geographic ranges appear likely to contain cryptic species and (2) the vast majority of subspecies diversity was overestimated, while the species diversity was underestimated. This inaccuracy may have negative consequences for conservation, and additional work is needed to refine the taxonomy of the group.

**Figure 1:**
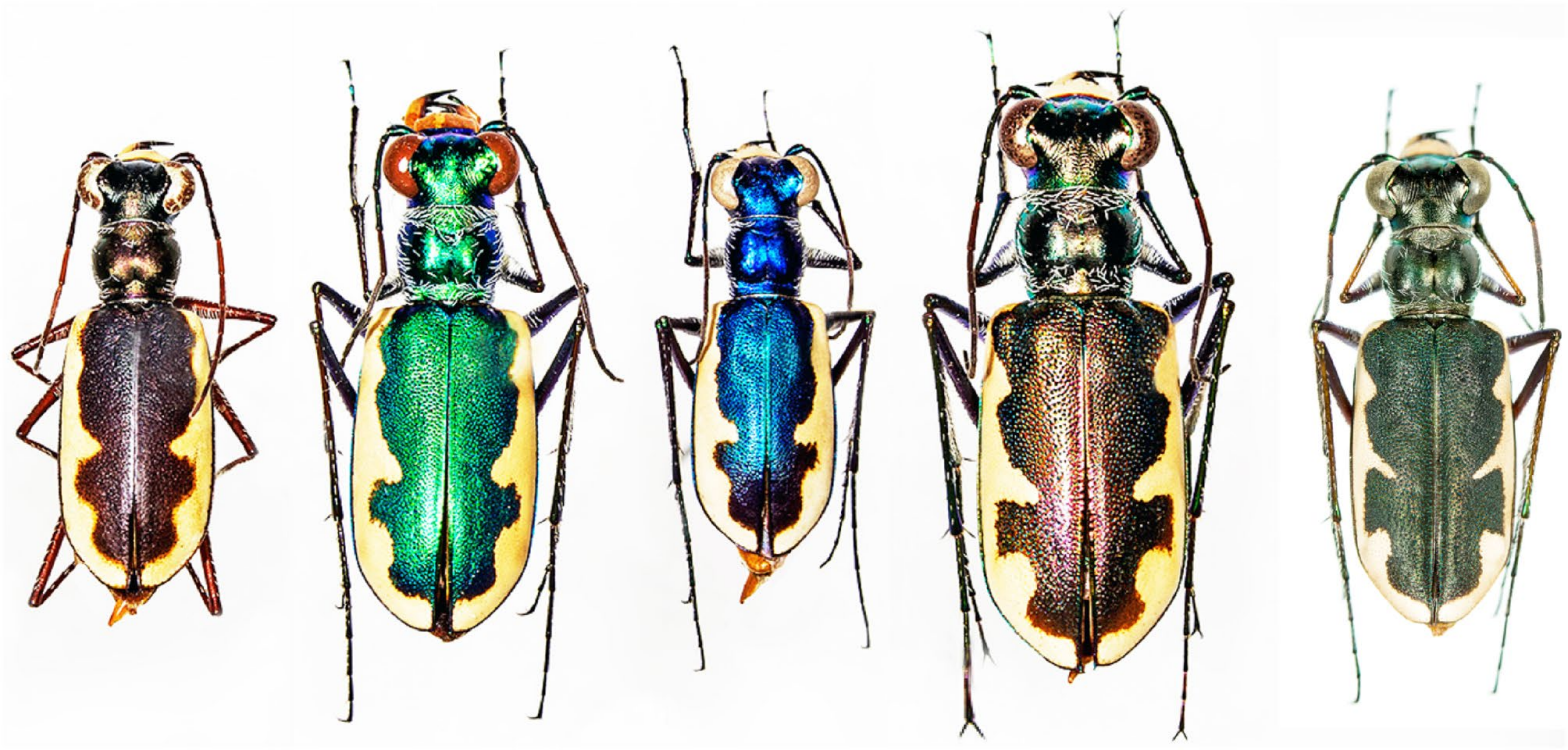
Morphological variants within the *Eunota circumpicta* species group. From left to right: *E. c. pembina* (North Dakota: Pembina County), *E. c. johnsonii* (Texas: Pecos County), *E. mecocheila* (Mexico: Coahuila), *E. c. circumpicta* (Texas: Kleberg County), *E. c. circumpicta*, unnamed variant from Houston area (Texas: Hardin County), described later in this paper.

**Figure 2:**
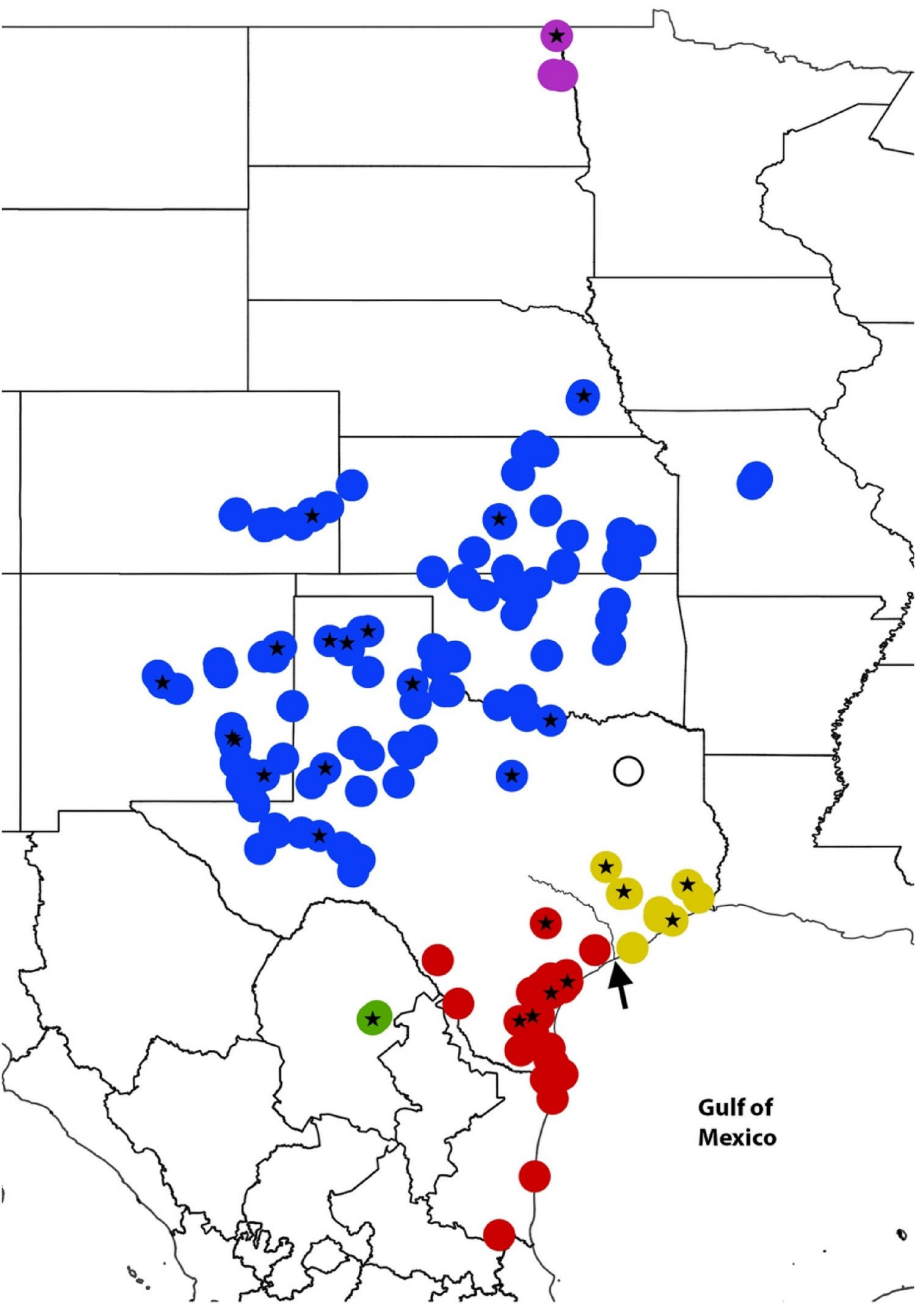
Map of known *E. circumpicta* species group localities. Stars represent populations that were sampled for mtDNA and genomic analyses. Colors correspond to morphologically distinct taxa and variants. Purple = *E. c. pembina*, blue = *E. c. johnsonii*, green = *E. mecocheila*, red = *E. c. circumpicta* (typical), yellow = smaller, duller *E. c. circumpicta* from Houston area. The open circle represents a putative population from Grand Saline, TX that is based on a single worn specimen of uncertain affinity and was not available for DNA work. The arrow points to the mouth of the Colorado River (i.e. Matagorda Bay), a putative barrier (see “Discussion”). Figure created with QGIS 3.34 (https://qgis.org/).

The species *Eunota circumpicta* (LaFerté-Sénectère, 1841) (Fig. 1) is a habitat specialist in alkali/saline flats and beaches and has a historically wide geographic range in North America, from northern Mexico to North Dakota, central New Mexico to central Missouri (Fig. 2). Some isolated geographic populations and named subspecies are rare (e.g., *E. circumpicta pembina* from North Dakota) or possibly extirpated (e.g., *E. circumpicta johnsonii* from central Missouri), and as such, this species group may be of great conservation concern. As with most tiger beetle species that possess a large geographic range, there are phenotypic variants with respect to body color and the white markings on their fore wings (called *maculations*) that have been used to describe several subspecies. First, the nominate subspecies, *E. circumpicta circumpicta* (LaFerté-Sénectère, 1841), is a mostly coastal lineage found along the Gulf Coast area of Texas and Mexico. However, populations of *E. c. circumpicta* along the upper Texas coast (specifically, northeast of the Colorado river and Matagorda Bay) are considerably smaller with duller elytral texture, subtly different maculations, and different predator escape behaviors, which has been hypothesized by the third author (SJR) to represent a distinct taxon. Second, *E. circumpicta johnsonii* (Fitch, 1856) is an inland subspecies found in northern and western Texas, New Mexico, Oklahoma, Colorado, Kansas, Nebraska and Missouri. Third, *E. circumpicta pembina* (Johnson, 1993) is a subspecies isolated in northeastern North Dakota, approximately 800 km from the nearest known population of *E. c. johnsonii*. Fourth, collecting efforts in the 1990s led to the discovery of an apparently disjunct population initially believed to be *E. c. johnsonii* from Coahuila, Mexico, but recent integrative taxonomy work by Duran & Roman^51^ determined that this population represented a distinct species based on morphology, genetic and biogeographic data and was recently named *E. mecocheila* Duran & Roman, 2021. Lastly, other geographic populations have been hypothesized to represent additional subspecies and some had been previously named, such as *E. circumpicta salinae* (Vaurie, 1951) from Nebraska, based on slight differences in average body length and coloration. However, this hypothesized taxon is generally regarded as a synonym of *E. c. johnsonii* by most workers.

In the present study, we tested these morphologically based taxonomic hypotheses using multilocus genomic and mtDNA analyses. Consistent with other recent work on tiger beetle taxonomy, we found multiple cryptic species within the previous taxonomic concept of *Eunota circumpicta*, some of which were previously recognized as subspecies. We found that the mtDNA and genomic datasets did not identify the same taxonomic units and that the mtDNA genealogy was incongruent with all other genetic and morphological patterns. Overall, we were able to make suggestions for testing species and subspecies validity.

## Materials and methods

### Specimen collection

Historical localities for the *E. circumpicta* species complex were obtained from published records and publicly available online data sources, including iNaturalist, BugGuide and Symbiota Collections of Arthropods Network (SCAN). Collecting efforts were conducted to include all nominal subspecies and as many geographic areas as possible (Fig. 2). All specimens and their collection localities are indicated in Supplemental Information (Table S1).

### Molecular sampling, mtDNA

Field collected specimens (preserved directly in ~ 96% ethanol) and when possible, pinned specimens, were sampled for molecular data. Non-destructive DNA extractions were performed as in Duran et al.^42^ to preserve whole specimens for morphological observations. All specimens were sampled for both mtDNA and subsequent genomic analyses.

We used the CB1 and CB2 primers^52^ to amplify a 424 bp region of the mitochondrial cytochrome b gene (cytb). We chose this gene due to its short fragment length, which would be more likely to amplify from degraded DNA from old or pinned specimens. PCR conditions follow those of Laroche et al.^32^ Mitochondrial sequences were deposited in the NCBI GenBank Database under the accession numbers MZ404132–MZ404270 and GBS data was submitted to the NCBI Sequence Read Archive under accession numbers  SAMN39917255–SAMN39917382.

### Mitochondrial analysis

We inferred a mitochondrial genealogy with IQ-TREE v.1.6.9^53^. Model selection was performed using ModelFinder in IQ-TREE with the best model chosen using BIC^54^. The tree with the best maximum likelihood score was selected from 200 independent searches. For each of the 200 runs, we estimated nodal support using 1000 ultrafast bootstraps and 1000 SH-aLRT tests. We used the -bnni command to avoid severe model violation resulting in overestimation of nodal support when performing ultrafast bootstraps. DNaSP 6.12 was used to calculate pairwise divergences between taxonomic groups. Using the same mtDNA dataset, we then created a haplotype network using POPART 1.7^55,56^ to visualize the distribution of ancestral alleles (internal to the network) and derived alleles (tips of the network). Haplotype networks are frequently used to visualize the geographic arrangement of alleles in a way not captured by mtDNA gene genealogies. We used the Templeton, Crandall and Sing^57^ (TCS) method for inferring a network, as this has been used extensively with mitochondrial nucleotide sequence data to estimate relationships across a wide range of genetic divergence^58–60^. We also explored a Median Joining method for creating the haplotype network, which gave qualitatively similar results to the TCS method.

Mitochondrial DNA sequences were tested for selective neutrality via the following tests: dN/dS (ratio of non-synonymous substitutions to synonymous substitutions), Fu’s *F*^61^ and Tajima’s *D*^62^ using DNaSP 6.12. These tests were calculated for the entire *E. circumpicta* clade, as there was no taxonomic structure within the clade, and considerable haplotype mixing occurred amongst the subspecies and forms. Additional measures of population demographics, including haplotype diversity and nucleotide diversity, were calculated using DNaSP 6.12.

### Multilocus marker generation and analysis

A genotype-by-sequencing (“GBS”) approach was used to generate multilocus nuclear markers. Specifically, we used a restriction enzyme associated DNA sequencing (RADseq) procedure to produce reduced complexity libraries as described in Parchman et al.^63^ and utilized previously for tiger beetles^32,42,50^. RADseq libraries were sent for sequencing at the University of Texas Genomic Sequencing and Analysis Facility (Austin, TX). Sequencing was conducted in two parts. A primary sequencing run on an Illumina NovaSeq platform in a single SP lane produced 342,693,349 100 bp raw single end reads.

Ipyrad version 0.9.84 (https://ipyrad.readthedocs.io/) is a GBS toolkit for sequence assembly and analysis and was used to process all reads^64^. Reads from each sequencing run were demultiplexed separately to reduce the number of reads assigned incorrectly. Sequences then underwent an initial filtering step where any reads with more than five base calls that had a Phred-scaled quality less than 33 were removed. From here, data was divided into four subsets based on the analyses we planned to conduct as it has been shown that identifying SNPs independently in subsets of data for downstream use can reveal patterns of genetic differentiation that might otherwise go undetected if all data is processed together^65^. The first of these subsets was for RAxML phylogenetic analyses, which included all *E. circumpicta* group taxa^48,51,66^ and sampled populations (Fig. 2)—as well as three *E. fulgoris* (Casey, 1913) individuals and an *E. togata* (LaFerté-Sénectère, 1841) individual as outgroups. The next two subsets were informed by the results of the mtDNA genealogy and initial RAxML topology (below) and were as follows: (1) an inland clade including all *E. c. johnsonii* and *E. c. pembina*, and (2) a coastal clade of all *E. c. circumpicta*, including the eastern populations from Hardin, Galveston and Brazos Counties (herein “Houston area”) hypothesized to be an undescribed lineage. For each of these subsets, loci were identified and filtered. Loci that were present in fewer than four samples, had more than 20% SNPs, or more than 8 indels were removed to exclude poor alignments. Individuals in each subset that recovered fewer than 2000 loci were removed from the dataset with the exception of the dataset used for RaxML as this program has been shown to be robust to the type of missing data generated in RADseq^67^. Across all subsets, individuals had data from an average of 21,988 loci.

### Multilocus nuclear trees generated from SNP data

A maximum likelihood tree was constructed from all SNP data from 125,895 total loci using RAxML 8.2.12^67^. The concatenation of all SNPs into a single matrix, without subsampling from each locus, is a common practice when generating trees from RADseq data through RAxML^68–71^. This analysis included 58 individuals, including those from every population and taxonomic group analyzed in this study. We performed N = 100 bootstrap analyses followed by 10 rapid hill-climbing maximum likelihood searches from random trees utilizing the GTRGAMMA substitution model^72^. All other parameters were left as default.

### Principal component analysis of SNP data

The ipyrad analysis toolkit was used to conduct all principal component analyses. Prior to these analyses, data sets underwent additional filtering to minimize missing data: loci that were present in less than 50% of individuals in each taxonomic group or present in less than 75% of individuals overall were excluded. Remaining missing data was imputed using an algorithm in ipyrad that randomly sampled genotypes based on the frequency of alleles within each taxonomic group. For loci with multiple SNPs, a single SNP was randomly subsampled during each replicate analysis to reduce potential effects of linkage on results^73^. Analyses were each repeated 25 times and the centroid of all points from each sample was plotted.

### Bayesian clustering analysis using SNP data

We used STRUCTURE v.2.3.4 to perform unsupervised assignment of individuals to K populations using a Bayesian clustering algorithm^74^. The model identifies population membership of individuals based on patterns of linkage and Hardy–Weinberg disequilibrium. As with the Principal Component Analyses, loci that were present in less than 50% of individuals in each taxonomic group or present in less than 75% of individuals overall were excluded from each subset of data analyzed. STRUCTURE was run with values of K ranging from K = 2 to K = 6. Values of K were run with 250,000 burn-in steps and 250,000 calculation steps and replicated 10 times with default parameters. For loci with multiple SNPs, a single SNP was randomly subsampled during each replicate analysis to reduce potential effects of linkage. We ran analyses under the no admixture model, as all morphologically distinct forms were geographically allopatric. Additionally, analyses were run with an admixture model. Results were qualitatively similar and we present the results with no admixture. Plots of mean log probability and delta K values of each model were used to identify optimal K values^75^. For both of the major clades (identified from the multilocus tree), K = 2 was a direct test of the taxonomic hypotheses that each clade contained two species, based on morphology, and these plots are presented below. Models for each additional value of K are presented for a more comprehensive view of the genetic variation among populations^65,76,77^ (see Supplemental Figure S1).

### Estimating genetic distance using SNP data

Genetic distance between populations, measured as Latter’s F_ST_^78^, were estimated by the hierfstat R package Version 0.5-11. Population designations and comparisons were made between the following groups based on the collective results of RAxML phylogeny, PCAs and STRUCTURE analyses: *E. mecocheila* and all *E. circumpicta* populations; *E. c. johnsonii* + *E. c. pembina* and *E. c. circumpicta* west of Matagorda Bay + *E. c. circumpicta* (?) from Houston area; *E. c. johnsonii* and *E. c. pembina*; *E. c. johnsonii* “*salinae*” from Nebraska and all other *E. c. johnsonii* populations.

### Species delimitation using SNP data

The BEAST (v2.7.3) package SPEEDEMON (v1.1.0) was used as a further measure of species delimitation^79^ on the subset of individuals used for RAxML phylogenetic analyses excluding the *E. fulgoris* and *E. togata* outgroups (N = 69). Multilocus SNP data for these 69 individuals was subset with Plink (v2.00a3.3) for unlinked SNPs present in at least 90% of individuals, yielding a matrix of 343 SNPs with very little missing data. SPEEDEMON’s Yule Skyline Collapse model was employed, where samples with an estimated ancestral species time below a threshold, epsilon, are collapsed into a single species. Epsilon values of 10^–3^ and 10^–4^, within the range of values found to be effective in Douglas and Bouckaert^79^, were tested with an MCMC chain of ten million and all other model parameters left as default.

## Results

### mtDNA analyses

**Figure 3:**
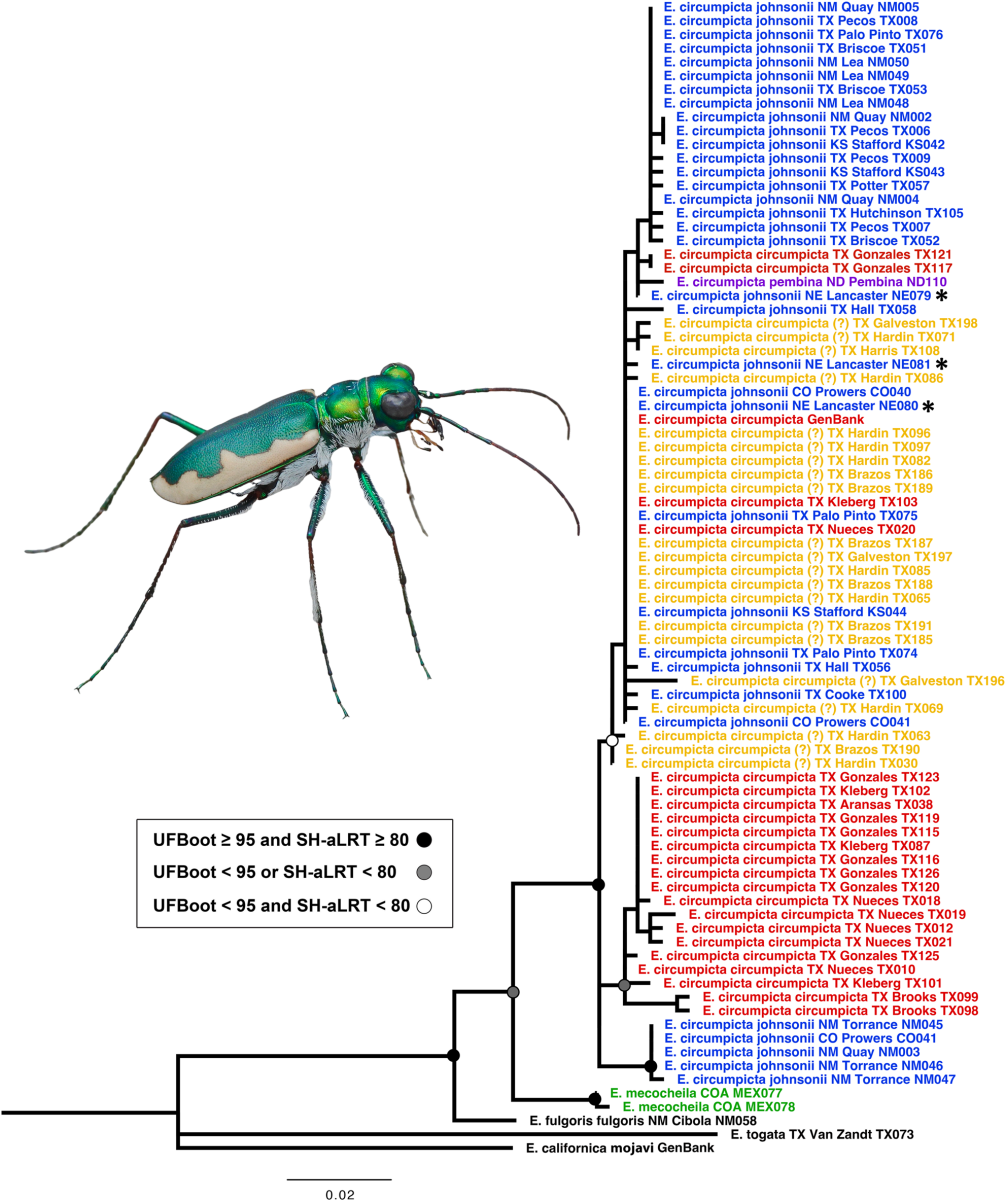
Maximum-likelihood mtDNA genealogy inferred in IQ-TREE based on cytb gene. Taxon naming follows previous conventions^48,51,66^, and colors refer to the same entities as in Fig. 1. *E. c. circumpicta* (yellow) from Houston area have not been previously named as a distinct taxon. Asterisks indicate individuals that have been called *E. c. salinae*, generally regarded as a northern population of *E. c. johnsonii*^48,66^.

The mitochondrial genealogy (Fig. 3) recovered a monophyletic *E. circumpicta* species group (*E. mecocheila* + *E. circumpicta*) with a deepest split between *E. mecocheila* and *E. circumpicta* (3.3% average pairwise distance). Beyond this, branch lengths were very short and there was little structuring according to subspecies/morphology, or geography, broadly. Some subclades in the tree were geographically constrained to local/regional areas, but overall, there was mixing of mtDNA haplotypes from taxonomic/geographic groups throughout the topology.

**Figure 4:**
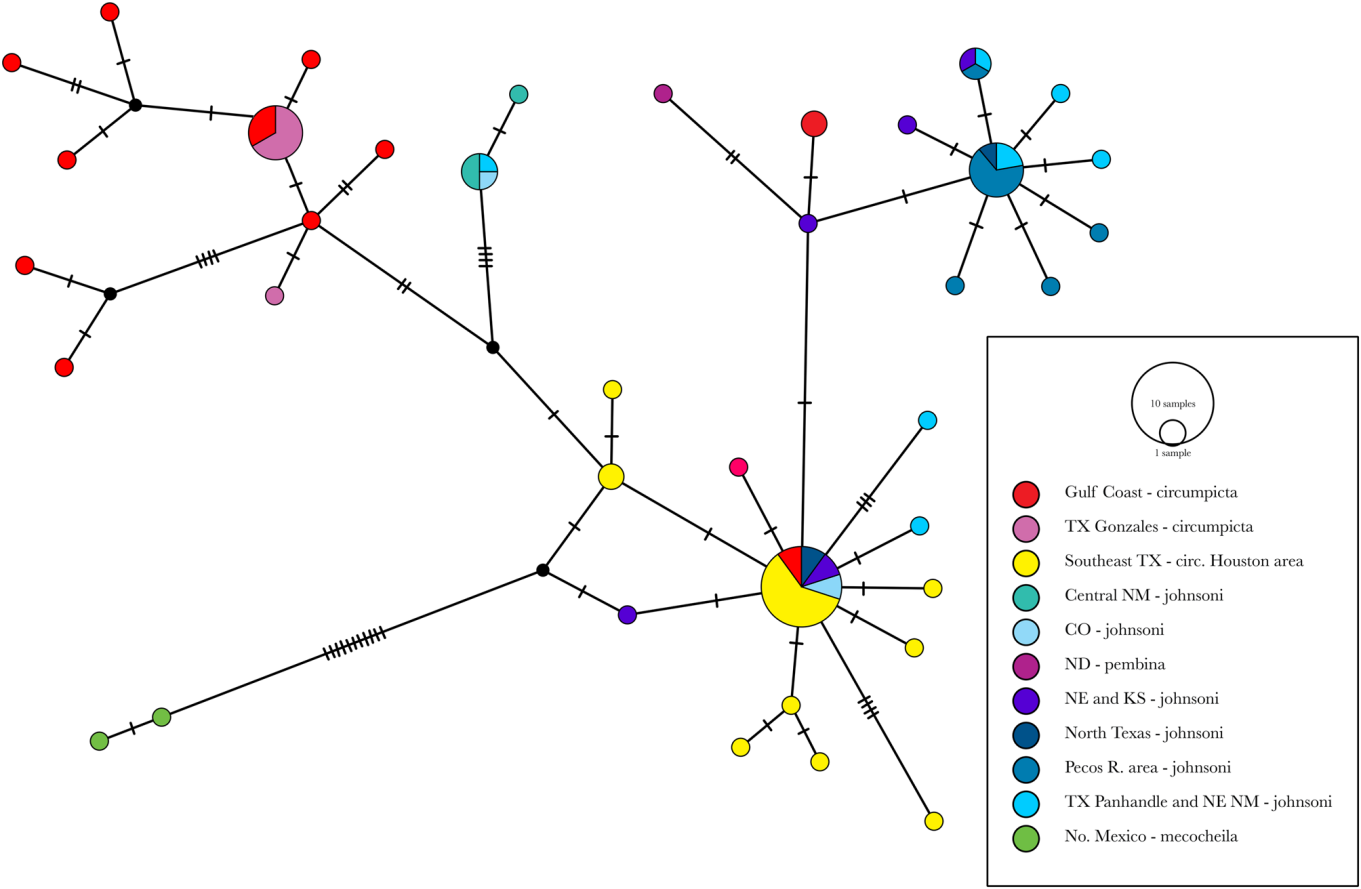
TCS haplotype network generated with POPART 1.7^55^ shows the evolutionary relationships of the cytb sequences in this study. Each hatch mark indicates a mutational step between adjacent alleles. The color of each circle corresponds to the geographic location of the sequence (see inset figure legend). The size of the circle is proportional to the haplotype frequency.

The TCS haplotype network (Fig. 4) indicated that *E. mecocheila* and *E. circumpicta* were separated from each other by 14 mutational steps, the largest subdivision within the network. Within *E. circumpicta*, there was no clear geographic pattern to the distribution of ancestral or derived alleles, with internal/ancestral alleles predominantly found in southeastern TX, but also in areas much farther west and north, such as Nebraska and Colorado. Some of the most derived alleles were found in the western Gulf Coast and west Texas/New Mexico area surrounding the Pecos River.

Neutrality tests of *E. circumpicta* mtDNA were as follows: the dN/dS ratio of non-synonymous to synonymous substitutions was very low and significantly deviating from expectations of neutrality (dN/dS = 0.014; P < 0.01). Fu’s *F* and Tajima’s *D* were both negative at − 24.021 and − 3.282, respectively, and both significantly deviating from neutrality, P < 0.01. These tests collectively demonstrated that the pattern of mtDNA variation in the *E. circumpicta* group departed significantly from neutral expectations. Haplotype diversity was high (0.909) and nucleotide diversity was low (0.0093).

### Maximum likelihood phylogeny from genome-wide SNP data

**Figure 5:**
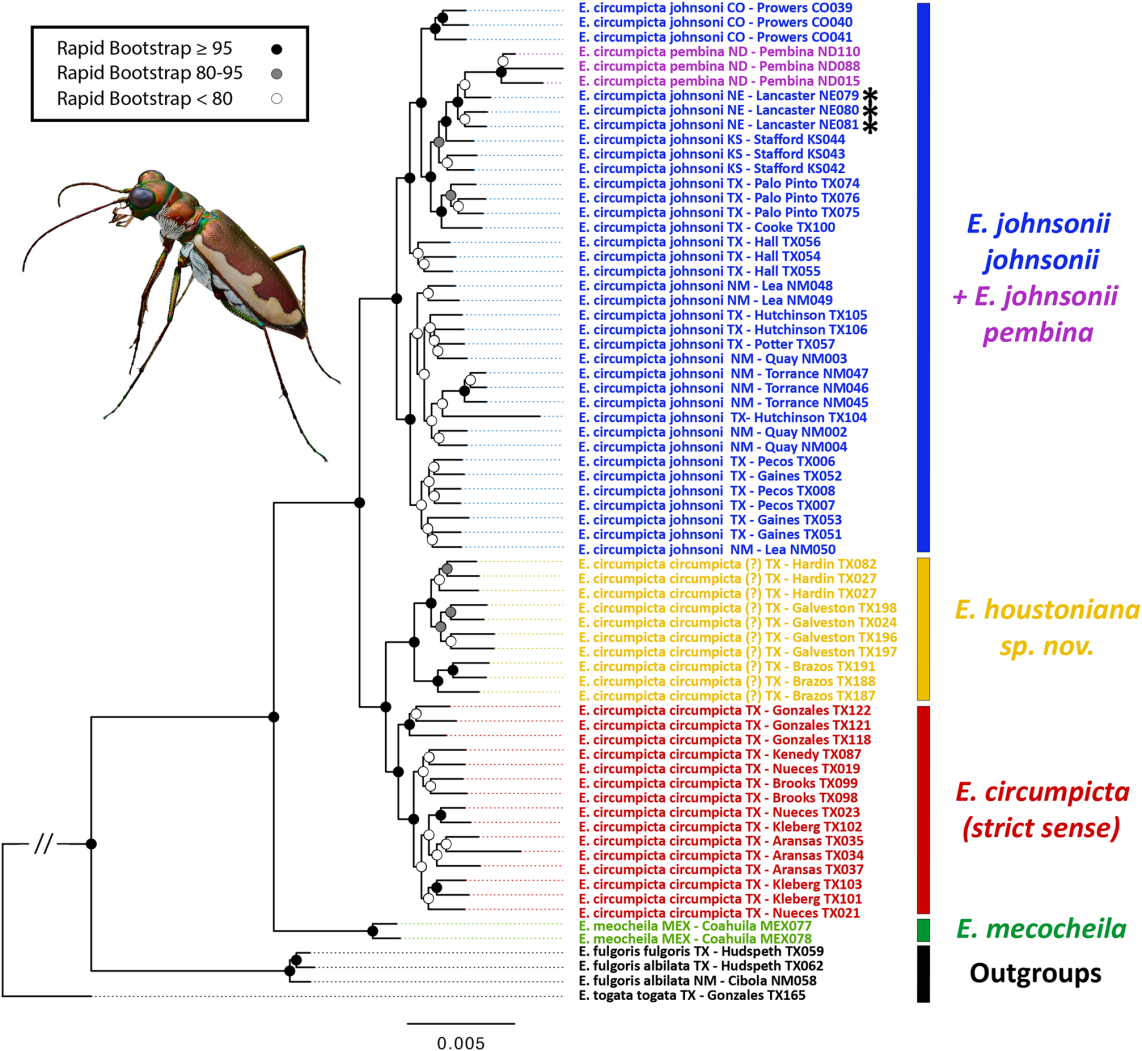
Maximum likelihood (RAxML) tree based on SNP dataset. Topology based on 125,895 total loci. Taxon naming follows previous naming conventions^48,51,66^ with colors highlighting taxonomic groupings, as in Figs. 2 and 3. Asterisks denote individuals belonging to *E. c. salinae*, generally recognized as a synonym of *E. c. johnsonii*^48,66^. Vertical bars indicate final taxonomic groups identified by the conclusion of this study, based on the plurality of results (see “Taxonomy” section).

The RAxML tree based on 125,895 SNP loci (Fig. 5) recovered a monophyletic *E. circumpicta* species group (*E. mecocheila* + *E. circumpicta*) with a major split between *E. mecocheila* and *E. circumpicta*, as also observed in the mtDNA genealogy. The topologies differed beyond this. Dissimilar to the mtDNA tree, there was clear structuring by geography and taxonomy/morphology within *E. circumpicta*. The first split within *E. circumpicta* was between *E. c. johnsonii* + *E. c. pembina* and typical *E. c. circumpicta* + *E. c. circumpicta* Houston area.

Herein, we will refer to these two major clades as the Interior Clade and the Gulf Clade, respectively. Within the Interior Clade, *E. c. pembina* was monophyletic, but nested within a paraphyletic *E. c. johnsonii* clade, closest to northern *E. c. johnsonii* populations from Nebraska and Kansas. Within the Gulf Clade, there were two reciprocally monophyletic clades, corresponding to the typical *E. c. circumpicta* populations south and west of the Colorado River and Matagorda Bay and the Houston area *E. c. circumpicta* populations that have been hypothesized to represent a unique taxon. All of the aforementioned clades were supported by bootstrap values above 95.

### PCA from genome-wide SNP data

**Figure 6:**
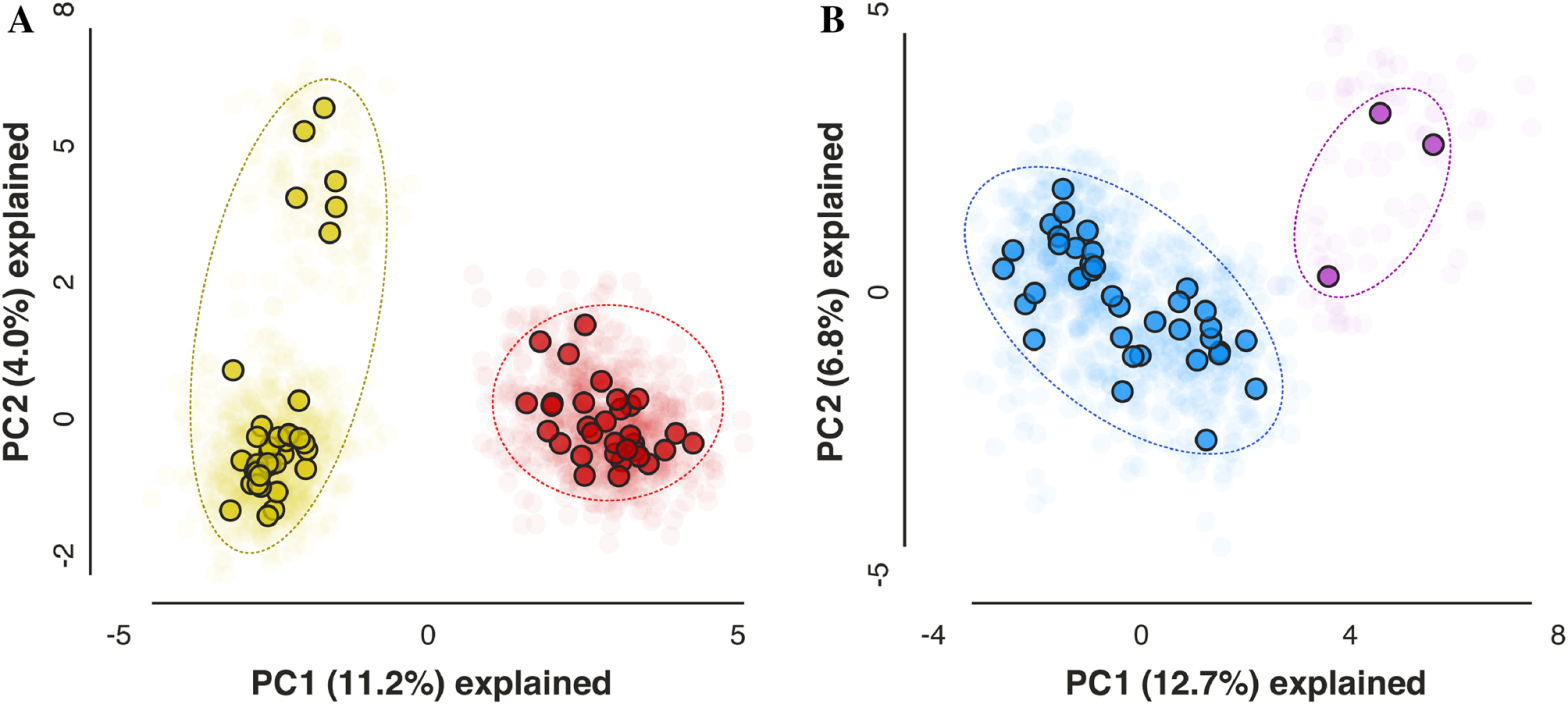
Principal component analyses (PCA) of 4126 (**A**) and 930 (**B**) SNPs for the Gulf and Interior Clades, respectively. Loci were limited to those found in a minimum of 50% of individuals in each nominal taxonomic group and in 75% of individuals overall to produce a SNP matrix with relatively little missing data (17.70%; 17.25% for **A** and **B** respectively). Transparent points represent replicate analyses (N = 25) while opaque points represent the centroids of these replicates. (**A**) PCA of Gulf Clade individuals. Red points on the right-hand side of the graph corresponds to typical *E. c. circumpicta* individuals from west of Matagorda Bay. Gold points on the left correspond to morphologically atypical *E. c. circumpicta* individuals from East of Matagorda Bay. (**B**) PCA of Interior Clade individuals. Blue points correspond to *E. c. johnsonii*, purple points correspond to *E. c. pembina*.

A principal component analysis of the SNP data was conducted to assess the clustering of individuals within each of the two deeply separated clades, the Interior Clade (i.e., *E. c. johnsonii* and *E. c. pembina*) and the Gulf Clade (i.e., typical *E. c. circumpicta* and *E. c. circumpicta* from the Houston area) (Fig. 6A,B). For the Interior Clade, the first two principal components explained 12.7% and 6.8% of the total variation in the dataset. The observed results indicated a separation between the *E. c. johnsonii* and *E. c. pembina* individuals. For the Gulf Clade, the first two principal components explained 11.2% and 4.0% of the total variation in the dataset. The largest separation was between typical *E. c. circumpicta* and *E. c. circumpicta* from the Houston area. Within the Houston area clade, there was considerable separation between the northern population from Brazos County reflecting their isolation on an inland geologic feature called a salt dome (top of PC2) and the other Houston area populations on coastal saline soils or coastal salt domes (bottom of PC2). Salt domes are a dome-shaped structures in sedimentary rocks formed where a large mass of salt has been forced upward in the bedrock, which can influence surface soil salinity.

### Bayesian clustering analysis using genome-wide SNP data

**Figure 7:**
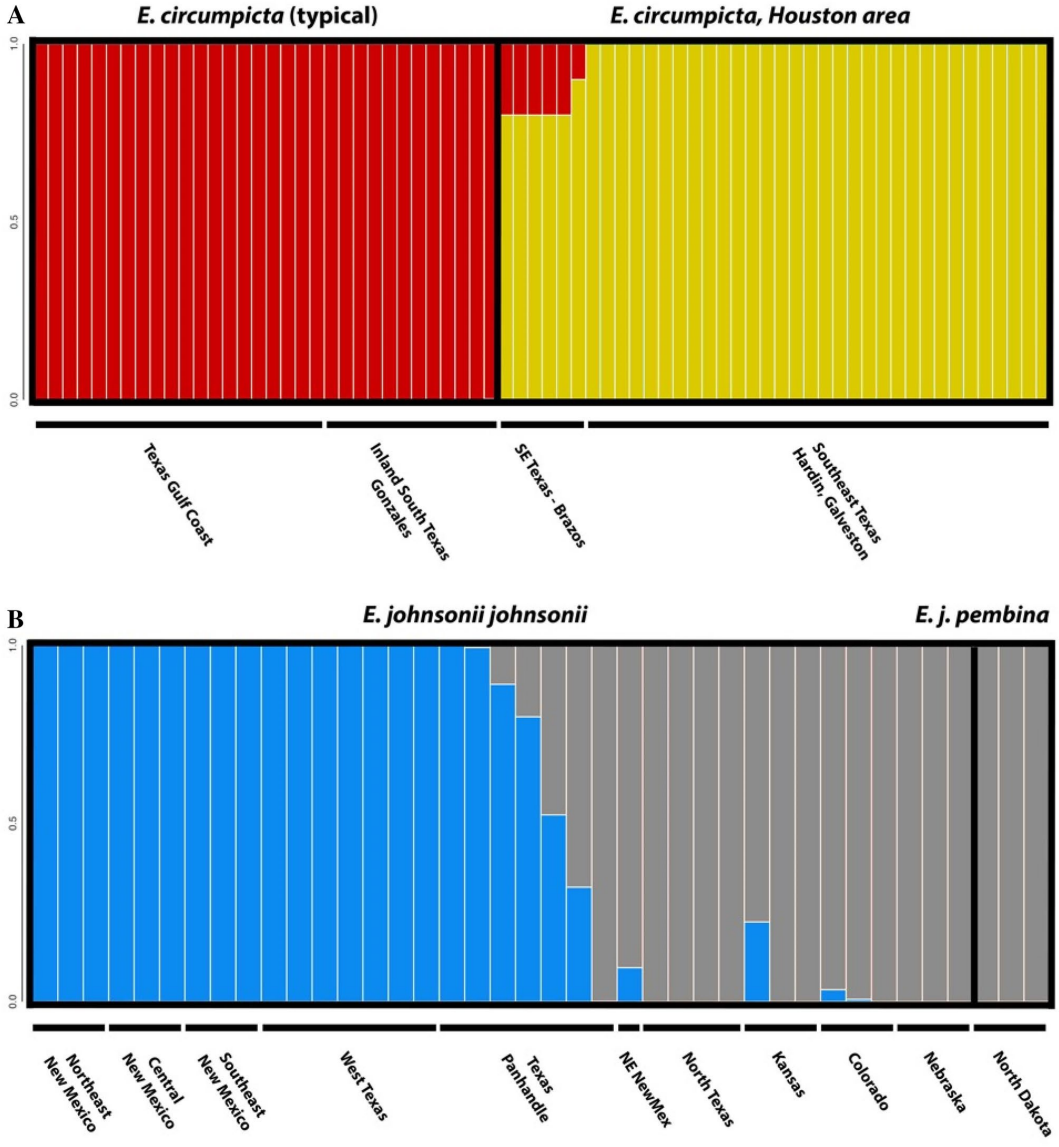
STRUCTURE analyses of (**A**) the Gulf Clade based on 4106 SNPs. Shown is K = 2, a test of the hypothesis that the Gulf Clade represents two taxa, as predicted based on morphology. Additional K runs are reported in Supplemental Materials. (**B**) Interior Clade based on 882 SNPs. Shown is K = 2, a test of the hypothesis that the Gulf Clade represents two taxa, as predicted based on morphology and historical taxonomic treatments. Additional K runs are reported in Supplemental Materials.

Based on the results of the multilocus phylogeny and PCAs, we conducted a set of Bayesian clustering analyses using the program STRUCTURE v.2.3.4 to assess the genetic structuring within the Gulf and Interior Clades (Fig. 7). Within the Gulf Clade, at K = 2, the two groups identified corresponded to the populations of *E. c. circumpicta* (typical morphology) south and west of the Colorado River and Matagorda Bay and the morphologically and behaviorally distinct Houston area populations. At all additional K populations, the identified groups did not change significantly. Within the Interior Clade, *E. c. johnsonii*, *E. c. pembina* and *E. c. salinae* were never recovered as distinct in the STRUCTURE analyses under any K scenarios.

### Genetic distance estimates

Based on the SNP dataset, Latter’s F_ST_^78^ was calculated as follows: F_ST_ = 0.306 between *E. mecocheila* and the rest of the taxa (Gulf and Interior Clades); F_ST_ = 0.213 between the Gulf and Interior Clades; F_ST_ = 0.195 between typical *E. c. circumpicta* west of Matagorda Bay and morphologically atypical *E. c. circumpicta* from Houston area; 0.062 between *E. c. johnsonii* and *E. c. pembina*; and 0.048 between *E. c. salinae* and *E. c. johnsonii*.

### Species delimitation using genome-wide SNP data

SPEEDEMON identified a species topology with 81.79% posterior support. This topology included five species: *E. mecocheila*, typical *circumpicta*, Houston-area *circumpicta*, *johnsonii* and *pembina*. The other species topology recovered had 18.21% posterior support, and included four species: *E. mecocheila*, typical *circumpicta*, Houston-area *circumpicta* and *johnsonii* + *pembina*. Both supported topologies recovered the same taxa with the exception of *pembina* that was included with *johnsonii* in the second topology.

### Taxonomy

**Figure 8:**
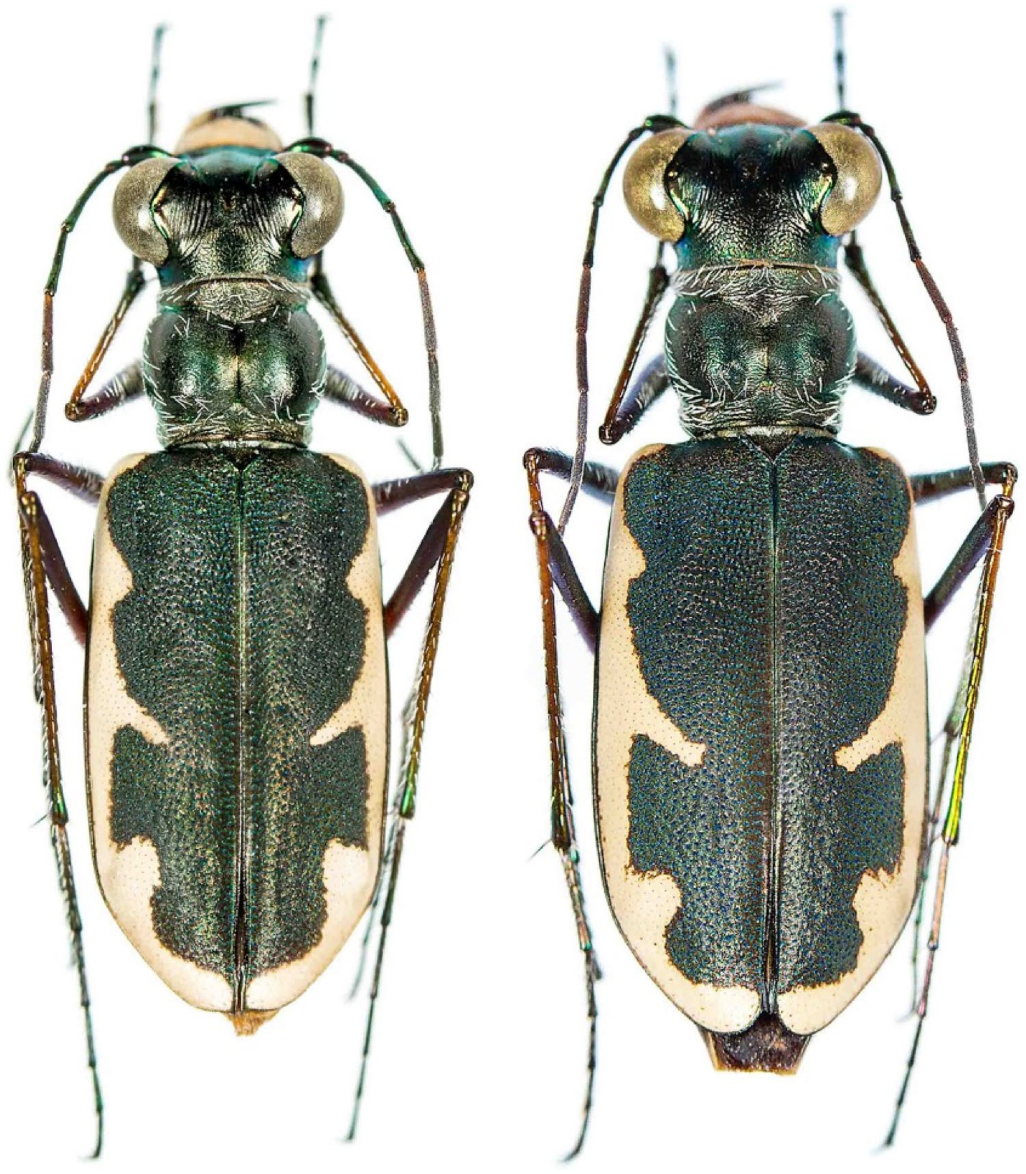
Dorsal habitus of male and female *Eunota houstoniana*, sp. nov. from the type locality: Texas: 1.5 km north of Sour Lake.

Given the plurality of results summarized in Table 1, including reciprocal monophyly, considerable genetic divergence between the major clades based on 125,895 loci, morphological and/or behavioral distinctiveness and support from Bayesian species delimitation methods^79^ we recognize the following taxonomic entities: (1) *E. mecocheila* remains as a distinct species endemic to Coahuila, Mexico, as previously assessed^51^; (2) *E. c. johnsonii* encompassing the entire Interior Clade is elevated to a full species, *E. johnsonii*; (3) *E. johnsonii* is recognized as having two subspecies, *E. j. johnsonii* from North Texas west to New Mexico and Colorado and north to Nebraska, and the geographically isolated *E. j. pembina* from North Dakota (see “Discussion”); (4) from the Gulf Clade, *E. c. circumpicta* is elevated to a full species, *E. circumpicta*, to include all populations west of the Colorado River and Matagorda Bay, and (5) north and east of that area, a new species, *E. houstoniana* sp. nov. (Fig. 8) is recognized and formally described in this paper.

**Table 1:**
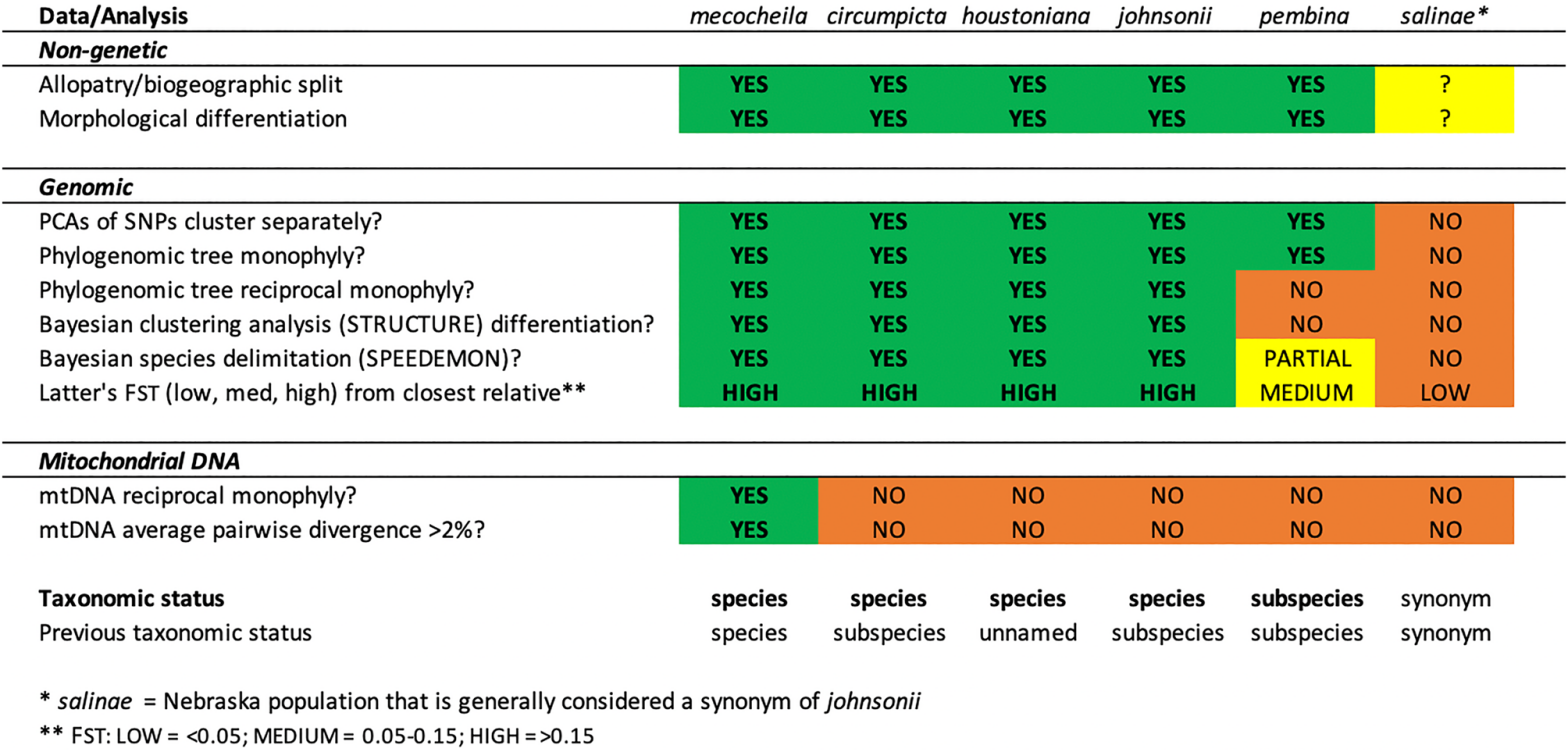
Support for taxonomic hypotheses in the *E. circumpicta* group. Based on the results, populations of *E. c. circumpicta* east of Matagorda Bay are named as a new species in this paper, *E . houstoniana* **sp. nov.**

The following taxa are formally recognized:

*Eunota circumpicta* (LaFerté-Sénectère 1841), stat. nov.

*Eunota johnsonii johnsonii* (Fitch, 1856), comb. nov.

*Eunota johnsonii pembina* (Johnson, 1993), comb. nov.

*Eunota mecocheila* Duran & Roman, 2021

**Figure 9:**
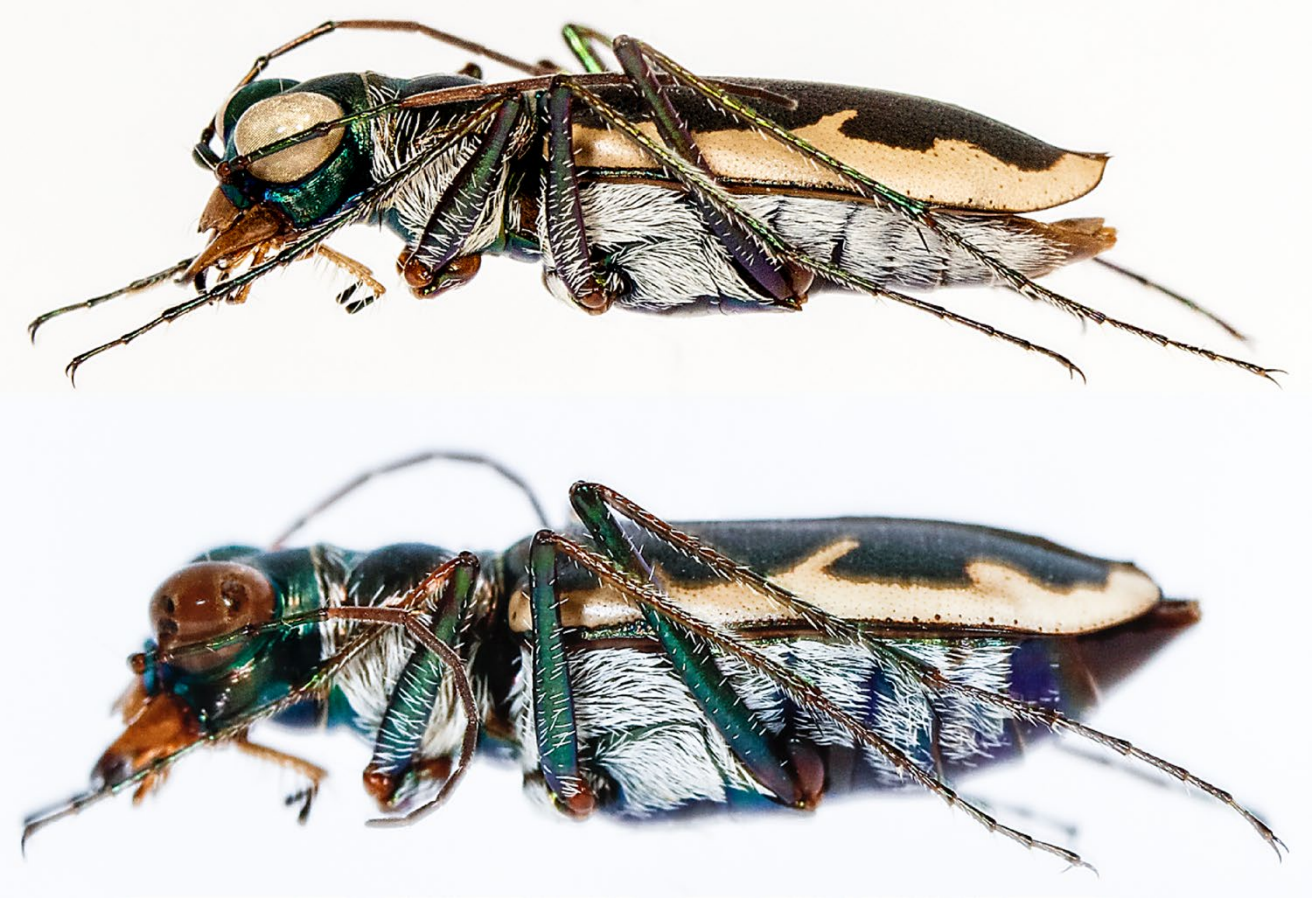
Lateral habitus of *E. houstoniana*, sp. nov. male (top), female (bottom).

**Figure 10:**
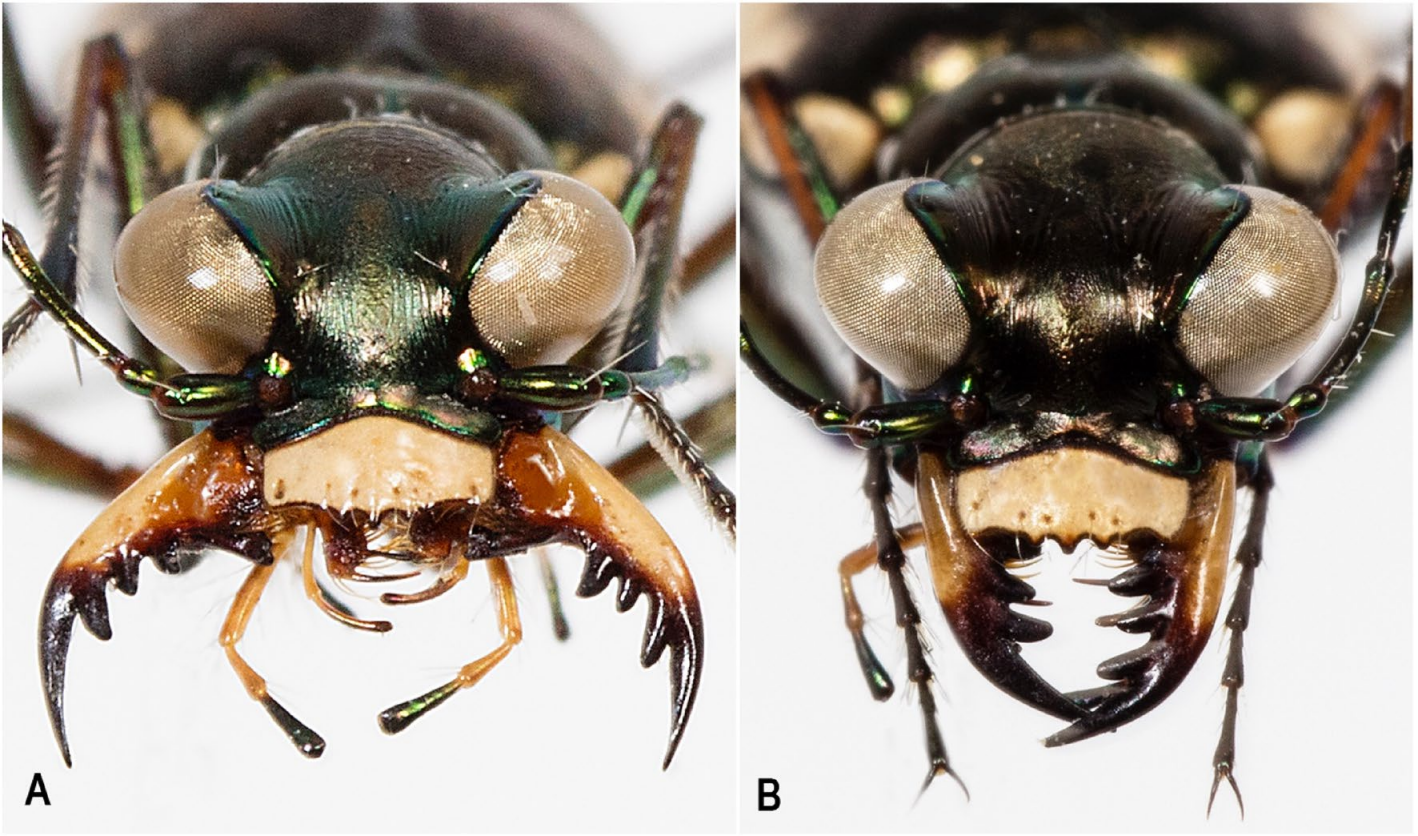
Frontal habitus of *E. houstoniana*, sp. nov. (**A**) male, (**B**) female.

**Figure 11:**
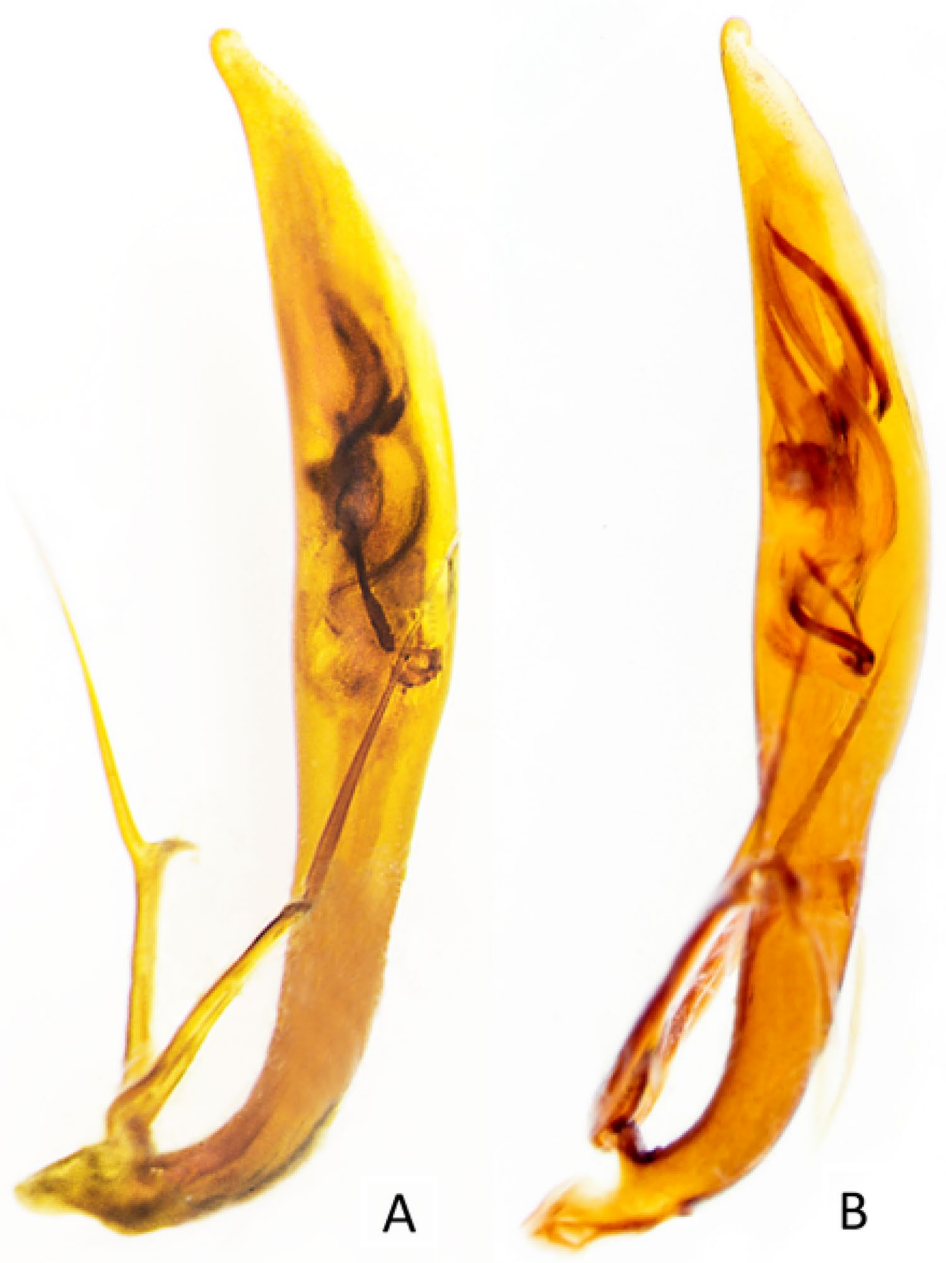
Aedeagus of (**A**) *E. houstoniana*, sp. nov., (**B**) *E. circumpicta*.

*Eunota houstoniana* Duran, Roman, Bull, Herrmann, Godwin, Laroche & Egan 2024, sp. nov. (Figs. 8, 9, 10, 11A). See following description

Description of *E. houstoniana*, sp. nov.

This new taxon is registered with ZooBank:

urn:lsid:zoobank.org:act:B80C5729-EF33-48B6-A607-745341FFF0B8

Specimens of a putatively undescribed *Eunota* similar to *E. circumpicta* were collected by the third author (SJR) and David Brzoska on June 6, 1987, 1.5 km north of Sour Lake, TX. The third author observed that this population consisted of significantly smaller individuals and possessed a different elytral texture and maculation patterns. Behaviorally, the Sour Lake individuals were observed to run into vegetation or fly a very short distance (1–5 m) to escape, in contrast to typical nominate *E. circumpicta* individuals that typically fly a considerable distance (frequently 50–100 m) and almost never land in vegetation. No additional populations of this atypical form were discovered until the authors of this paper searched new localities based on knowledge of underground salt domes, which influence ground-level soil salinity, and through the use of aerial photographs, supplemented by a thorough search of additional collection records from nearby areas, (e.g. SCAN-bugs.org, iNaturalist, private collections, USNM).

The plurality of genetic analysis support the delineation of this *Eunota* as separate from nominate *E. circumpicta* (Table 1). This is congruent with morphological and morphometric data, as described below. Lastly, this species is supported as separate from *E. circumpicta* based on historical biogeography, (see “Discussion”).

A total of 113 specimens were available to be examined for morphological characters. The total body length excludes the labrum and is measured as the distance from the anterior margin of the clypeus to the elytral apex, including the sutural spine. The width of the pronotum is measured to include the lateral margins of the proepisterna. The width of the head is measured as the distance between the outer margins of the eyes.

All tiger beetles were measured using a Leica M125 Stereoscope using Leica Application Suite. Lighting was provided by a Leica LED5000 MCI with Rotterman contrast TM Transmitted Light Base with Rotterman contrast TM, brightfield and two-sided darkfield. Two body measurements were taken, an elytra measurement and a full-length measurement. In addition to measurements of the total body length and elytral length, foveal density was measured under higher magnification. Due to an apparent difference in foveal density between those present in maculations and those present on the rest of the elytra, each was measured separately. The photos were taken into photoshop and a grid (0.087 mm × 0.087 mm) was overlaid. Then, 6 grid squares in both the maculation and just the elytra were chosen arbitrarily around the photograph and fovea were counted.

Specimen images were captured by means of a Canon EOS 1D Mark IV camera with a Canon 100 mm macro lens. Images were montaged and edited with Adobe Photoshop CS6. Scale bars and measurements were calibrated with an ocular micrometer on Olympus SZ61 and SZX7 microscopes. The final digital images were processed with Adobe Photoshop CS6.

Type material is deposited in the following institutional and private collections (acronyms used in the text are in parentheses): National Museum of Natural History, Smithsonian Institution, Washington, DC, USA (NMNH), Texas A&M Collection (TAMUIC), Sam Houston State Natural History Museum (SHNHM), Collection of Stephen J. Roman (SJRC), Collection of Daniel P. Duran (DPDC), Collection of John A. Shetterly (JASC), Collection of C. Barry Knisley (CBKC), Collection of Jason P Schmidt (JPSC).

### *E. houstoniana*, sp. nov. type material

HOLOTYPE: 1m#, TEXAS: Hardin Co. // 1 mi N Sour Lake // June 6, 1987 // Coll: S.J. Roman (USNM). PARATYPES: 2m#, 2f#, TEXAS: Hardin Co. // 1 mi N Sour Lake // June 6, 1987 // Coll: S.J. Roman (USNM); 2m#, 2f#, TEXAS: Hardin Co. // 1 mi N Sour Lake // June 6, 1987 // Coll: S.J. Roman (TAMUIC). 34m#, 30f#, TEXAS: Hardin Co. // 1 mi N Sour Lake // June 6, 1987 // Coll: S.J. Roman (SJRC); 1m#, 1f#, TEXAS: Hardin Co. // 1 mi N Sour Lake // June 6, 1987 // Coll: S.J. Roman (JASC); 1m#, 1f#, TEXAS: Hardin Co. // 1 mi N Sour Lake // June 6, 1987 // Coll: S.J. Roman (CBKC); 6m#, 6f#, TEXAS: Hardin Co. // 1 mi N Sour Lake // 17-June-2014 // Coll: D.P. Duran (DPDC); 7m#, 5f#, TEXAS: Hardin Co. // 1 mi N Sour Lake // Aug 10, 2012 // Coll: D.P. Herrmann (USNM); 1m#, TEXAS: Harris Co. // Warren Ranch Rd, 4 mi S. Hockley // Katy Prairie Conservancy // August 2021 // Coll: S. Egan (SHNHM); 4m#, 3f#, TEXAS: Brazos Co. // near Lick Creek Park // September 2, 2021 // Coll: S. Egan, W. Godwin (SHNHM); 1m#, 2f#, TEXAS: Galveston Co. // Bolivar Flats // GPS: 29.37981, -94.72909 // October 6, 2006 // Coll: J. Schmidt, J. Owens (JPSC). All type specimens labelled: HOLOTYPE or PARATYPE, respectively.

### Diagnosis

*Eunota houstoniana* sp. nov. (Figs. 8, 9, 10, 11A) can be distinguished from all other similar *Eunota* by the following combination of characters. Body length is 10.7–13.1 mm, mean 11.4 mm, ground color is olive green to dark olive-brown, maculations include a complete marginal line with a narrowly pointed short middle band, and lacks setae on the frons, genae and clypeus. The only species that could be confused with *E. houstoniana* sp. nov. is *E. circumpicta*. *Eunota circumpicta* maculations are similar to *E. houstoniana* sp. nov. but *E. circumpicta* middle band is less pointed and typically thicker at the base (Fig. 1).

### Description

Small to medium-sized *Eunota*. Body (Figs. 8, 9) length 10.7–13.1 mm, mean f# 11.6 mm, mean m# 10.8 mm. Head (Figs. 8, 10) noticeably wider than pronotum due to large eyes, width 2.8–3.5 mm, mean f# 3.3 mm, mean m# 2.9 mm, head concolorous with pronotum and elytra, typically olive green to dark olive-brown; all head portions glabrous except for two supraorbital setae next to each eye. Frons slightly convex in median area, clearly delimited from clypeus, gradually blending into vertex. Frons surface with distinct longitudinal striae especially in lateral areas bordering eyes, vermiculate-striate in median area. Genae bright polished with deep longitudinal striae abruptly ending at border of vertex. Clypeus irregularly wrinkled to finely vermiculate. Labrum typically with 6–8 setae, ochre-yellow to pale yellow with thin dark brown to black border; male labrum tridentate, somewhat convex, length 0.7–1.1 mm, width 1.3–1.6 mm; female labrum tridentate, somewhat convex, length 0.8–1.1 mm, width 1.4–1.9 mm. Mandibles medium-sized, ochraceous, dark testaceous along edges. Maxillary palpi mostly yellow with metallic reflections, apical segment dark shiny metallic green to purple. Labial palpi ivory to pale yellow, apical segment dark metallic green to violet. Antennae of normal length, reaching humerus to basal third of elytron in female, to middle of elytra in male; scape with a single subapical seta; pedicel lacking any setae; flagellum antennomeres 3‒4 dark metallic and similar in color to rest of head with ring of apical setae and additional sparse setae throughout, antennomeres 5‒11 ochre-brown, dull-textured without metallic reflections and possessing erect setae in apical rings only, covered with fine pubescence throughout.

Pronotum (Fig. 8) 1.6–2.4 mm wide, mean f# 2.2 mm, mean m# 2.0 mm, length 2.1–2.6 mm, mean f# 2.5 mm, mean m# 2.2 mm, width to length ratio 0.55–0.70, slightly polished with metallic finish, color dark olive green to brown; sparse white decumbent setae present along marginal areas of dorsal surface, some individuals with additional white decumbent setae present along anterior and posterior margins; disc finely rugose to vermiculate with thin but distinct median line and strongly impressed anterior and posterior sulci; notopleural sutures clearly defined, not visible from dorsal view; proepisternum (Fig. 9) with decumbent white setae densely covering nearly the entire surface; all other ventral segments of thorax dark testaceous with metallic reflections, lateral areas covered in setae, median areas glabrous.

**Figure 12:**
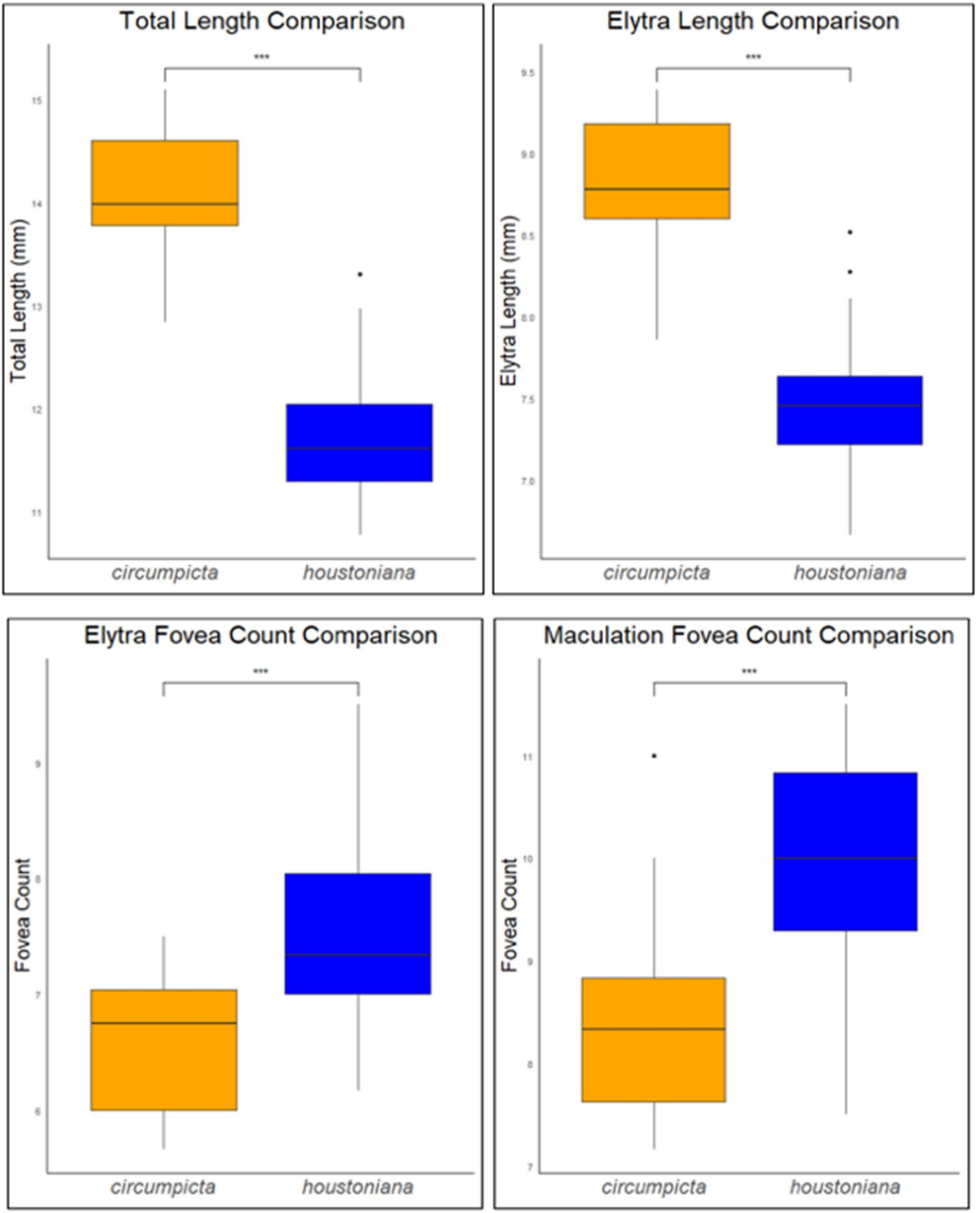
Boxplot comparisons of morphological differences between *E. circumpicta* and *E. houstoniana*. A two-tailed student’s t-test was used to test for statistical differences. ***A P value of < 0.001.

Elytra (Fig. 8) elongate, 6.6–8.2 mm length, mean f# 7.6 mm, mean m# 6.9 mm, shape similar in both sexes, but slightly wider in female, especially toward apical third; sutural spine small, fine microserrations present on elytral apices; elytra color dark green to olive brown, usually similar but not identical to color of head and pronotum; elytra slightly polished with dense punctures. Subsutural foveae present, but indistinct due to the background punctate texture; Elytral foveal density is greater than that of *E. circumpicta* (Fig. 12). Elytral maculations present, with a complete marginal band, a humeral lunule, pointed narrow partial middle band, and apical maculation.

Procoxae and mesocoxae dark testaceous with metallic blue to violet reflections, covered in dense setae; metacoxae dark testaceous with metallic green to violet reflections, nearly glabrous, possessing only a few setae along lateral margins; pro- and mesotrochanters with a single subapical seta, metatrochanters glabrous; femora metallic, with color similar to that of head and pronotum, femoral surface with rows of erect white setae dorsally and ventrally; tibiae mostly colored similarly to femora, clothed with white setae that are sparser and shorter than those of the femora; tarsi colored similarly to the tibiae, first three dilated protarsomeres in male with dense greyish-white setal pads.

Abdominal ventrites 1‒6 dark testaceous with most surfaces covered by metallic reflections; dense white decumbent setae present mostly along lateral third of each ventrite, except ventrite 6 in male and ventrites 5 and 6 in female, which are nearly glabrous. Aedeagus (Fig. 11A) shares similarities with *E. circumpicta* (Fig. 11B), possessing an elongate helical flagellum (Rivalier 1954). The middle/basal third of the *E. houstoniana* sp. nov. aedeagus appears more slender and parallel sided compared to E. circumpicta, which bulges slightly in the middle.

### Etymology

*Eunota houstoniana*, sp. nov. is named for the geographical proximity of all known localities to the Houston metro area.

### Distribution and habitat

*Eunota houstoniana,* sp. nov. is currently known only from nine localities in the southeastern portion of the state of Texas (Fig. 2). Beetles were collected in partially open salt pans surrounded by grasses and other vegetation. The species has been observed from early June to early October.

## Discussion

### Discordance between mtDNA and genomic analyses

The mtDNA genealogy (Fig. 3) and haplotype network (Fig. 4) were found to be at odds with the phylogenomic tree topology (Fig. 5) and all other genomic analyses (Figs. 6, 7). Topologies derived from mitochondrial markers may be inconsistent with those based on the rest of the genome^80–82^. This mitonuclear discordance can result from a number of reasons including incomplete lineage sorting, hybridization and introgression, sex-biased dispersal, or genetic sweeps from natural selection^83,84^. Mitonuclear discordance has complicated studies of species delineation^50,85–89^ and in the majority of these studies, morphology, life history or other non-genetic characters are more concordant with patterns inferred from the nuclear genome than from mtDNA. Although mtDNA is frequently used for species delimitation i.e., “DNA barcoding”^90–94^ there are substantial concerns about the utility of mtDNA as an indicator of evolutionary history. Inferences based on mtDNA alone can be unreliable, as mtDNA may fail to accurately delimit closely related species^50,85,88^. Moreover, because mtDNA may not be selectively neutral, this can be problematic for inferences about evolutionary history and estimates of divergence times between lineages^84^.

Our mitochondrial analyses recovered the deepest split (3.3% average pairwise divergence) observed between *E. mecocheila* and the rest of the *E. circumpicta* group, consistent with the phylogenomic tree. However, within the latter mtDNA clade there was no structuring by taxonomic groups or geography. The lack of structure in the *E. circumpicta* (broad sense) clade contrasts greatly with the phylogenomic tree, morphology and geography. One possible explanation is the purging of genetic variation due to purifying natural selection on mtDNA^95^. We conducted several tests of neutrality on this clade, including Fu’s *F*, Tajima’s *D* and the d_N_/d_S_ ratio (non-synonymous to synonymous substitutions). Tajima’s *D* is based on the allele frequency distribution of segregating nucleotide sites. A positive value indicates a bias towards intermediate frequency alleles, while a negative value indicates a bias towards rare alleles. Fu’s *F* is similar but based on the distribution of haplotypes. These neutrality tests departed significantly from neutral expectations (all at *P* < 0.01). The results of the Fu’s *F* and Tajima’s *D* were both negative (*F* = − 24.021, *D* = − 3.282) and the dN/dS ratio was less than one (0.014). All of these results were consistent with purifying or stabilizing selection on the cytb gene in the mtDNA. Besides purifying selection, an alternative explanation for the aforementioned mtDNA nucleotide and haplotype patterns is recent population expansion. This scenario seems unlikely, however, as the results of the genomic analyses demonstrated that there is considerable population structure within this *E. circumpicta* group clade, and the morphologically distinct lineages are all allopatric and monophyletic, supporting a scenario of historical isolation. Therefore, the removal of genetic variation in the mtDNA (cytb gene) due to purifying selection appears to be the most consistent with the plurality of data.

Had the mtDNA genealogy been used to delimit species (e.g., employing a method like Pons et al.)^91^, it would have lumped all of the *E. circumpicta* group taxa except *E. mecocheila* and this could have serious negative consequences for conservation planning. Two of the five taxa, *E. houstoniana* and *E. johnsonii pembina*, are geographically restricted and under threat of habitat loss due to development in these areas and E. *johnsonii pembina* is very rare and isolated to only two or three remaining metapopulations^16^.

### Concordance and discordance between different genomic analyses

Genomic analyses provided differing levels of support for the taxonomic hypotheses being tested (Table 1). One question is how should these different results be interpreted, and when discordance is observed between them, how to weigh each? Below we consider each type of genomic test, ordered by the depth of time at which one would expect to observe divergence between taxa.

Reciprocal monophyly has frequently been used as a criterion for delimiting species and evolutionarily significant units (ESUs)^36,96^. Reciprocal monophyly may be expected to take typically 9–12 *N* (effective population size) generations to be reached at 95% of nuclear loci^97^. Insect effective population sizes are typically much larger than vertebrate animals and may be large even in geographically fragmented insect species of conservation concern^98^. Therefore, reciprocal monophyly may require considerable time and a lack of reciprocal monophyly is expected when populations or species have diverged more recently. In the *E. circumpicta* species complex, reciprocal monophyly in the nuclear phylogeny was observed for four of the six taxonomic hypotheses (Fig. 5): *E. mecocheila*, *E. circumpicta* (strict sense), *E. houstoniana* and *E. johnsonii*. *Eunota j. pembina* was monophyletic but nested within a paraphyletic *E. johnsonii* clade. It is not surprising that this set of populations would not exhibit reciprocal monophyly, as the area of North Dakota where it occurs was recently glaciated during the Last Glacial Maximum, ~ 11,000 years ago. The taxon known as *E. salinae*, which has generally been treated as a synonym of *johnsonii*^48,66^ due to weak morphological differentiation (alleged slight differences in color and average body size), was not reciprocally monophyletic from geographically proximate populations of *E. johnsonii*.

Bayesian species delimitation methods, such as those used in this study (SPEEDEMON v.1.1.0)^79^, are based on the multispecies coalescent model^99^, and do not require reciprocal monophyly to delimit taxa. As such, these methods may delimit taxa that may have speciated more recently (i.e., in less than 9–12 *N* generations). SPEEDEMON identified best supported topologies in our study that included either four or five species. Both supported topologies recovered the same taxa, *E. mecocheila*, *E. circumpicta* (strict sense), *E. houstoniana* and *E. johnsonii*, with the exception of *E. j. pembina* which was included with *E. johnsonii* in one of the two topologies.

Bayesian clustering algorithms such as STRUCTURE are able to determine population structure by assigning individuals to groups based on their multilocus genotypes by minimizing Hardy–Weinberg and linkage disequilibria^74^. These methods are frequently used to detect population subdivisions within a species or species group and can also be used to identify hybridization and introgression when applying the admixture model. We analyzed the Gulf and Interior Clades separately, as they were separated by considerable genetic distance (Latter’s F_ST_ = 0.213) and were reciprocally monophyletic. Within the Gulf Clade, there were two putative taxa to be assessed, based on morphology. The expectation was that at K = 2, there would be separation into groups comprised of (1) *E. circumpicta* (strict sense) that are typical in morphology and geographically distributed in the area from west of Matagorda Bay along the Gulf Coast, south into Mexico, and (2) populations east of Matagorda Bay (Houston area) that were smaller, rougher in elytral texture and with different maculations, named *E. houstoniana* sp. nov. in this publication. This predicted pattern was observed (Fig. 7A). Interestingly, the population of *E. houstoniana* sp. nov. from Brazos County (furthest inland and west) was observed to possess a small proportion of the *E. circumpicta* genome. For the Interior Clade, two generally recognized, morphologically based taxa (i.e., the subspecies *johnsonii* and *pembina*) were hypotheses to be tested. At K = 2, there was no separation along these taxonomic lines, but instead the Interior Clade separated into a southern group that included *E*. *johnsonii* individuals from most of Texas and New Mexico and a northern group that included all other *E. johnsonii* and *E. j. pembina* individuals spanning Colorado, Kansas, Nebraska, and North Dakota. Moreover, there was a gradient of individuals whose population assignment was mixed where these two subdivisions were geographically proximate in northeast New Mexico and the panhandle of Texas. Finally, the taxon *E. salinae* from Nebraska was never recovered as a distinct population at any K from 2 to 6.

Principal component analyses of SNP data can be used to infer genetic affinities between geographic populations and individuals. This method may allow for fine scale population genetic structure to be detected, the result of demographic processes such as colonization, isolation, migration and admixture. PCA results from our study showed that individuals largely clustered by morphological/taxonomic groups (Fig. 6). In the Gulf Clade, *E. houstoniana* sp. nov. were well separated from *E. circumpicta* (strict sense) along PC1 Within the *E. houstoniana* sp. nov. cluster there was additional separation between the individuals from Brazos County, TX, and all others along PC2. In the case of the Interior Clade, *E. j. pembina* individuals clustered and were separate from other *E. johnsonii* populations along PC0. This differentiation between *pembina* and *johnsonii* was not observed in the Bayesian clustering analyses or the phylogenomic tree.

F_ST_ is a measure of the genetic distance between populations due to isolation and reduction in breeding. Values of F_ST_ range from 0 to 1, with 0 representing no genetic differentiation and 1 representing complete differentiation. According to Wright^100^, F_ST_ values of 0.00–0.05 are considered “little” genetic differentiation, 0.05–0.15 is “moderate” differentiation and 0.15–0.25 is “great” differentiation. Four of the six taxonomic hypotheses (*E. mecocheila*, *E. circumpicta*, *E. houstoniana*, *E. johnsonii*) were found to exhibit great F_ST_ differentiation from each other (Table 1), with *E. j. pembina* exhibiting moderate differentiation from *E. j. johnsonii*. *Eunota salinae* displayed little differentiation from other proximate *E. j. johnsonii* populations.

### Species and subspecies delimitation and taxonomic changes

Four of the six taxonomic hypotheses were supported by all the genomic analyses (Table 1). In each case, there was broad agreement with morphology, and in this species group, each taxon was observed to be allopatric. Moreover, the deepest split between the Gulf and Interior Clades mirrored the same deep phylogeographic subdivision seen in the *E. togata* species group^32^, another salt-loving (halophilic) tiger beetle complex distributed broadly in the same areas of North America and often found in the same localities as members of the *E. circumpicta* group. Maps of both species’ groups show populations along the Gulf Coast and in the southern Great Plains, with gaps over the central limestone regions of Texas. Coincidence of these gaps would be expected if absence of suitable habitat is serving as a barrier to dispersal. Another distinct biogeographic break occurred only in the Gulf coast clade of *E. circumpicta*. This break is also supported by the biogeography and geologic history of the region, where the Colorado river is spring fed and likely never went dry historically, limiting the presence of surface salt needed by these halophilic beetles, thus serving as a permanent barrier to dispersal and gene flow.

As all genomic analyses (i.e., reciprocal monophyly, Bayesian species delimitation, Bayesian clustering algorithms, PCA of SNPs, F_ST_ values) and morphology were concordant and indicated divergence, these four taxa were recognized as distinct species: *E. mecocheila*, *E. johnsonii*, *E. circumpicta* and *E. houstoniana*, sp. nov. The cytb gene in the mitochondrial DNA was selectively non-neutral, displaying a signature of strong purifying selection. The genealogy showed no divergence of three of the four aforementioned taxa, and this suggests that in this group, mtDNA may not be informative with respect to closely related species, as has been the case in other studies^85–89^. In a recent tiger beetle study, mtDNA (also the cytb gene) was informative as to species boundaries in the *E. togata* species group, but not informative in others (e.g. *Cicindelidia politula* species group)^50^. Mitochondrial DNA is observed to display para- and polyphyletic species genealogies approximately 1/3rd of the time^83^, especially in the closest related species, where this issue is may be most problematic^101^ due to phenomena that include incomplete lineage sorting, hybridization and introgression (even low levels) or selective non-neutrality. As such, we recommend putting a lower emphasis on mtDNA genealogies and always including at least some concordant data, such as nuclear markers. Strict DNA barcoding would have failed to delineate multiple species in the *Eunota circumpicta* species group.

In the case of *E. j. pembina*, there was morphological distinctiveness, geographic separation (this taxon is separated by 800 km from the nearest known population of *E. johnsonii* to the south) and some genomic analyses indicated a subtle degree of divergence. Specifically, the PCA showed separation from *E. johnsonii* populations, the Bayesian species delineation showed distinctiveness in some topologies, but not others, and the F_ST_ values indicated a moderate amount of differentiation (Latter’s F_ST_ = 0.062). Given the lack of reciprocal monophyly and a lack of separation in the STRUCTURE analyses, *pembina* is best treated as a subspecies of *E. johnsonii*.

The taxon called *E. salinae* has generally not been recognized as a valid subspecies as it was delineated based on minimal morphological distinctiveness (slight differences in color and size), however, it was a hypothesis to be tested. It is not very distant from the nearest Kansas populations of *E. johnsonii* (~ 100 km) and not more geographically separated than other groups of populations on the periphery of the range of *E. johnsonii* (e.g., Colorado populations) and these other similarly disjunct populations have not been considered subspecies historically. *Eunota salinae* was not found to be distinct in any genetic analysis. The F_ST_ value of separation between *E. salinae* and other *E. johnsonii* populations was very low (Latter’s F_ST_ = 0.048). As such, we do not recognize this named taxon as a valid entity. It remains a synonym.

### Combining analyses for integrative taxonomy

Given that different genomic tests may detect different degrees of divergence, a combination of these analyses may be most informative, and may allow for robust delineation of species and subspecies. Moreover, we place these genomic tests in the context of biogeography, ecology, morphology, and behavior. For example, with respect to distinct species, (1) reciprocal monophyly would be expected approximately 9–12 N generations after speciation^97^, (2) Bayesian species delimitation analyses may detect speciation even if reciprocal monophyly has not yet occurred and therefore may be more sensitive and (3) in addition, an increase in F_ST_ is expected as populations exhibit reduction in gene flow and become most differentiated as a result of speciation. In most cases, F_ST_ > 0.15 is considered “great” and this threshold would generally be met for different species within a genus^102^. For our study, we conservatively decided that if at least two of the three above genomic criteria are met, in combination with breaks in geography, morphology, and/or behavior, it was reasonable justification to delimit species. Although morphological differentiation is not always present and is not necessary to delimit species^32,42,90^, concordance of morphology and/or behavior with at least two of the three above criteria is a strong indication that speciation has occurred, as is the case in our study. Moreover, consideration of the opportunity for dispersal and gene flow based on biogeographic barriers also helps place our results and taxonomic decisions in context.

Distinct species may be geographically overlapping (sympatric) to non-overlapping in distribution (allopatric, as observed in this study), with expectations that most, but not all, recently speciated taxa are likely to be allopatric^6^. Subspecies are generally distributed allopatrically or partially overlapping (parapatric, e.g. caribou, juncos, rattlesnakes), and some degree of geographic separation is typically included in the definition of subspecies^103^. Nearly all definitions of subspecies explicitly or implicitly state that some degree of reproductive isolation and genetic differentiation must be occurring^104^ or they would not be biologically meaningful. One would not expect the above species-level criteria to be met in the case of subspecies, but some genetic differentiation should be observed. For example, with respect to subspecies, (1) there may be separation (complete or partial overlap) of different subspecies along one or more axes in a principal component analysis, (2) some degree of structuring in a Bayesian clustering algorithm. Here, we recognize separate subspecies when at least one of these criteria is met, but the criteria for species are not met, and the putative subspecies are parapatrically or allopatrically distributed.

### Historical biogeography and potential impacts on speciation

The apparent barrier to geneflow at the Colorado River separating *E. circumpicta* from *E. houstoniana* sp. nov. is a hypotheses that is supported by the distributions of the two species/genetic lineages. The Colorado arises in a multitude of limestone springs that drain out of the Edwards Aquifer. These waters originate in a massive carbonate plateau that lacks any geological source of salt, and members of the *E. circumpicta* group can only be found in saline areas, coastal or inland. The Colorado waters are also more constant during drought periods because groundwater makes a greater contribution than neighboring rivers that are dominated by meteoric waters. The Colorado River runs fresher and longer during droughts. When combined at the mouth with the Brazos, it builds delta faster. These factors combine to inhibit the formation of salt flat habitat. Furthermore, there is evidence that at times during the Pleistocene, the ratio of meteoric vs groundwater has fluctuated widely. During wetter glacial episodes, the rivers have been much larger because of increased meteoric water input. During dry interglacials the input of groundwater has dominated the Colorado while other rivers ran dry and permitted deep oxidation of sediments^105^.

The Colorado River is well recognized as a barrier to other taxa. The aquatic isopod, *Caecidotia reddelli* occurs in groundwater throughout East Texas and inexplicably halts at the Colorado River with a few isolated exceptions. The Colorado also influences the population genetics of troglobitic salamanders of the genus *Eurycea* and amphipods of the genus *Seborgia*^106^.

Tests of this biogeographic hypothesis may be accomplished by more sampling in favorable areas that lack specimen records. Collecting on salt flats of the Ingleside barrier in Calhoun County in the vicinity of the Texas Parks and Wildlife Powderhorn Ranch preserve should produce specimens allied with *E. circumpicta* (strict sense). The coastal areas immediately east of Matagorda, Texas, especially in the vicinity of the Big Hill Salt Dome need to be investigated. These populations should turn out to be allied with *E. houstoniana,* if the Colorado River is a barrier to geneflow.

### Conservation implications

The importance of delineating species as well as subspecies is critical for conservation efforts, and these are the units most commonly protected by the Endangered Species Act of 1973. Re-evaluation of classic taxonomy in different species groups has revealed that species diversity has often been underestimated. In our study, we identified five taxa that had been historically considered *E. circumpicta* (one of which was just recently upgraded to full species, *E*. *mecocheila*). Three of these taxa are rare or isolated and each is known from a very small number of populations, *E*. *mecocheila* is known from one location in Northern Mexico, *E. j. pembina* from northeastern North Dakota, and *E. houstoniana* sp. nov. from the Houston, Texas area. Much of the saline flat habitat for *E. j. pembina* is surrounded by agriculture and these salines are considered “waste” areas and some have been plowed (Wayne Anderson, pers. comm. 2024) and other habitat has been lost from encroachment by invasive plant species. *Eunota houstoniana* sp. nov. is found in a heavily developed, urbanized area, and of the nine populations discovered by our group (mostly from historic collections), only four populations may be extant. Habitat loss is a serious concern for these populations, as remaining natural areas are shrinking due to land development for housing or agriculture. Now that this set of populations has been demonstrated to represent a genetically and morphologically distinct species, we hope that it may be a candidate for protection. We followed the NatureServe protocol for conducting a conservation status assessment^107^, and calculated that *E. houstoniana* would be ranked G2 S2, meaning globally “imperiled” with extinction, and “imperiled” with extinction in the state of Texas (the only place it is known to occur).

An emergent issue of this section is how conservation biologists and other stakeholders might incorporate population genomic analyses into their own conservation projects. The answer is complicated, including new molecular techniques, potentially higher sequencing costs with high-throughput sequencing, and increased bioinformatics training and computation challenges with the data, but we refer readers to the rich literature on incorporating the techniques we use here in to analyses of non-model organisms (see Peterson et al.^108^; Andrews et al.^109^; and citations therein for detailed discussion of methodologies and caveats).

### Conclusions and future directions

In this study, we tested morphologically based taxonomic hypotheses in a group of tiger beetles using multilocus genomic and mtDNA analyses. Consistent with other recent work on tiger beetle taxonomy, we found multiple cryptic species, some of which were previously recognized as subspecies. We found that the mtDNA and genomic datasets did not identify the same taxonomic units, and that the mtDNA genealogy was not selectively neutral, reducing its utility as an evolutionary marker. Finally, we were able to show a worked example of how to apply different analyses to species and subspecies delineation. This research has led to the validation and discovery of taxa that should be considered high priorities for conservation efforts.

This work reveals that additional unanswered questions exist, and that more work is needed to characterize the distribution of the group. For example, a single worn museum specimen from northeastern Texas (Van Zandt County) revealed that a population may exist or have existed in an area that could represent yet another new taxon, as it was geographically isolated in an inland saline area and morphologically ambiguous. Collection of fresh material would allow for molecular analyses. As these inland saline habitats are frequently considered “waste” areas, they are especially at risk of being lost to development. Already, it would appear as if the unusual Missouri populations of *E. johnsonii* are extirpated (T. MacRae pers. comm. 2020) as all known habitat has been lost to cattle grazing, changes in hydrology and invasive plants.

### Supplementary Information

Supplementary Information 1.

Supplementary Information 2.

## Data Availability

The datasets generated during and/or analyzed during the current study are available in the following repositories: NCBI GenBank Database under the accession numbers MZ404132-MZ404270 and GBS data was submitted to the NCBI Sequence Read Archive under accession numbers SAMN39917255–SAMN39917382.
