# Supplementary figures and images for: Species delimitation, discovery and conservation in a tiger beetle species complex despite discordant genetic data
Source: Sci Rep. 2024 Mar 19;14:6617. doi: 10.1038/s41598-024-56875-9 (PMC10951344; doi:10.1038/s41598-024-56875-9)

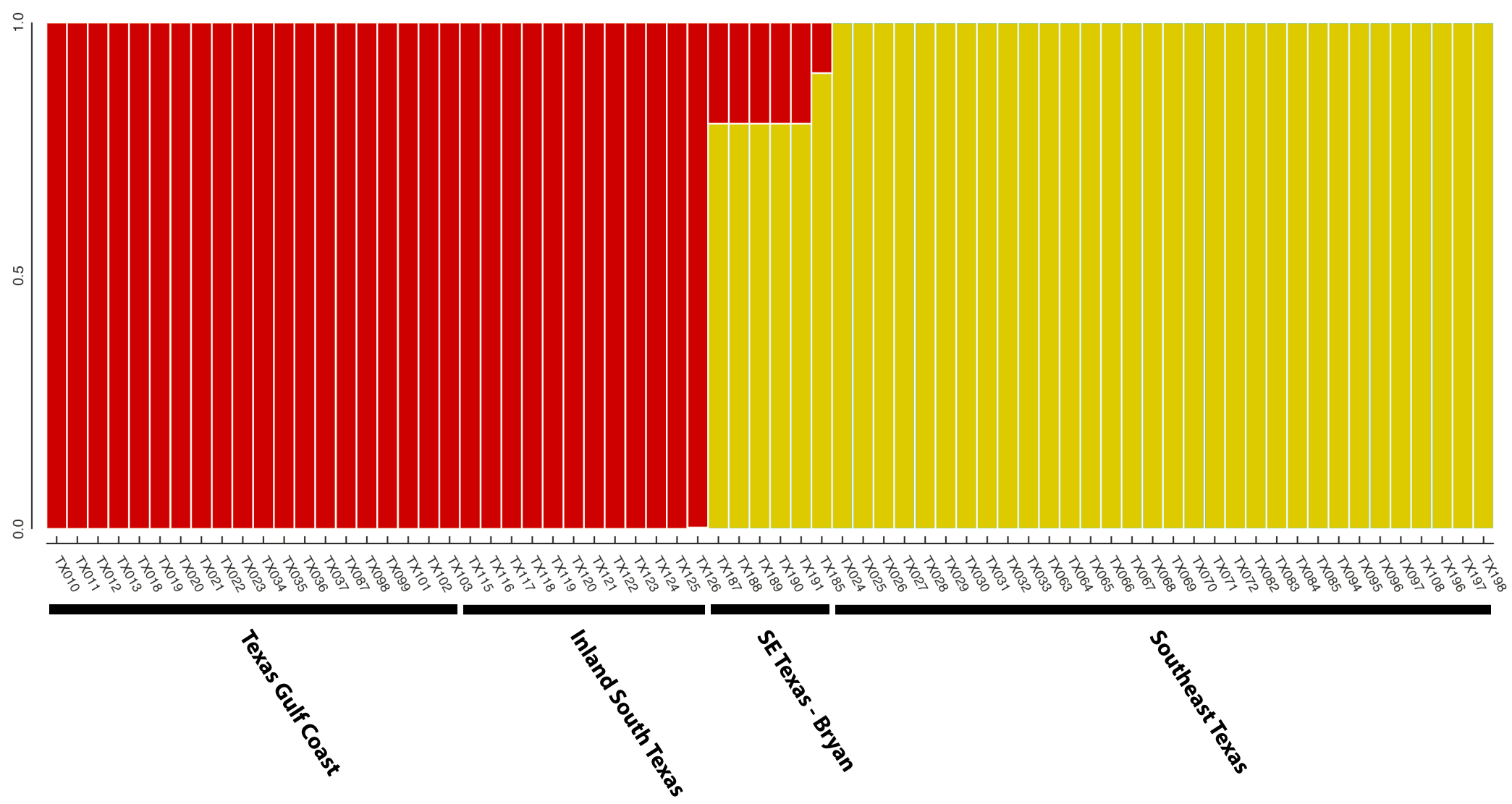

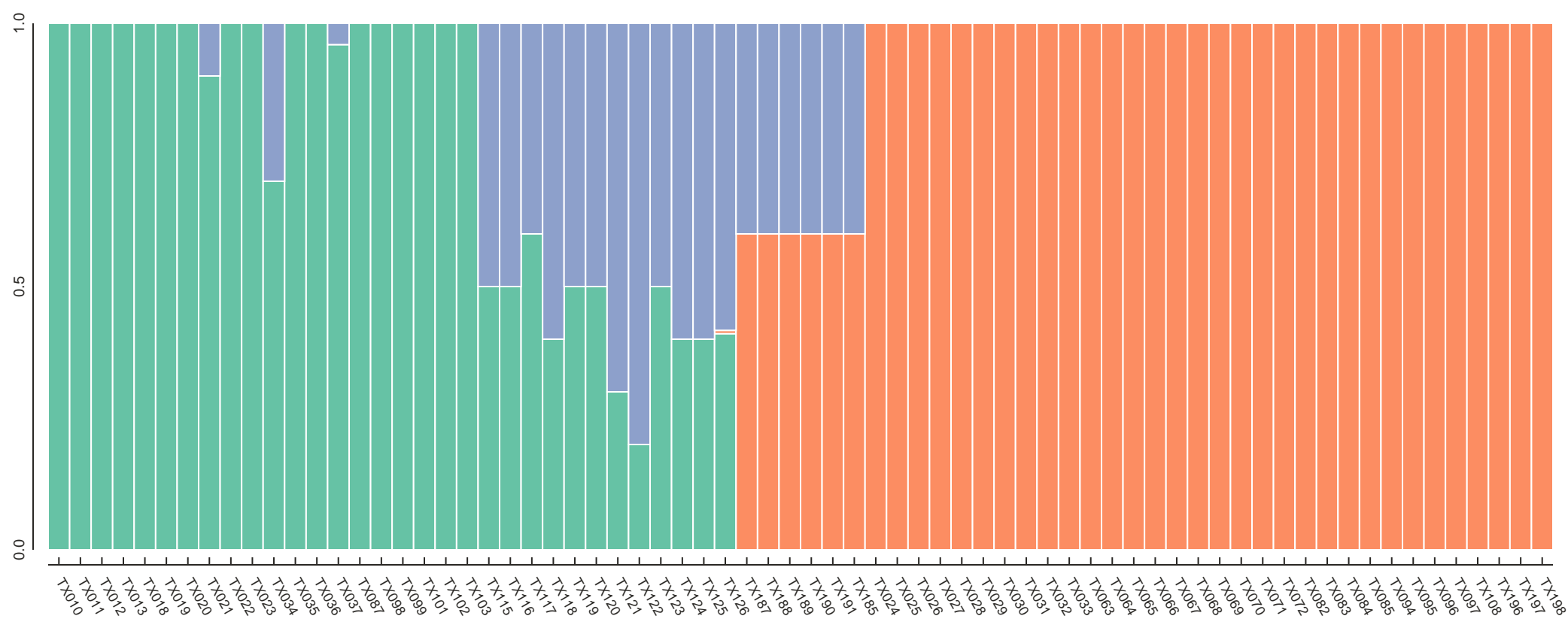

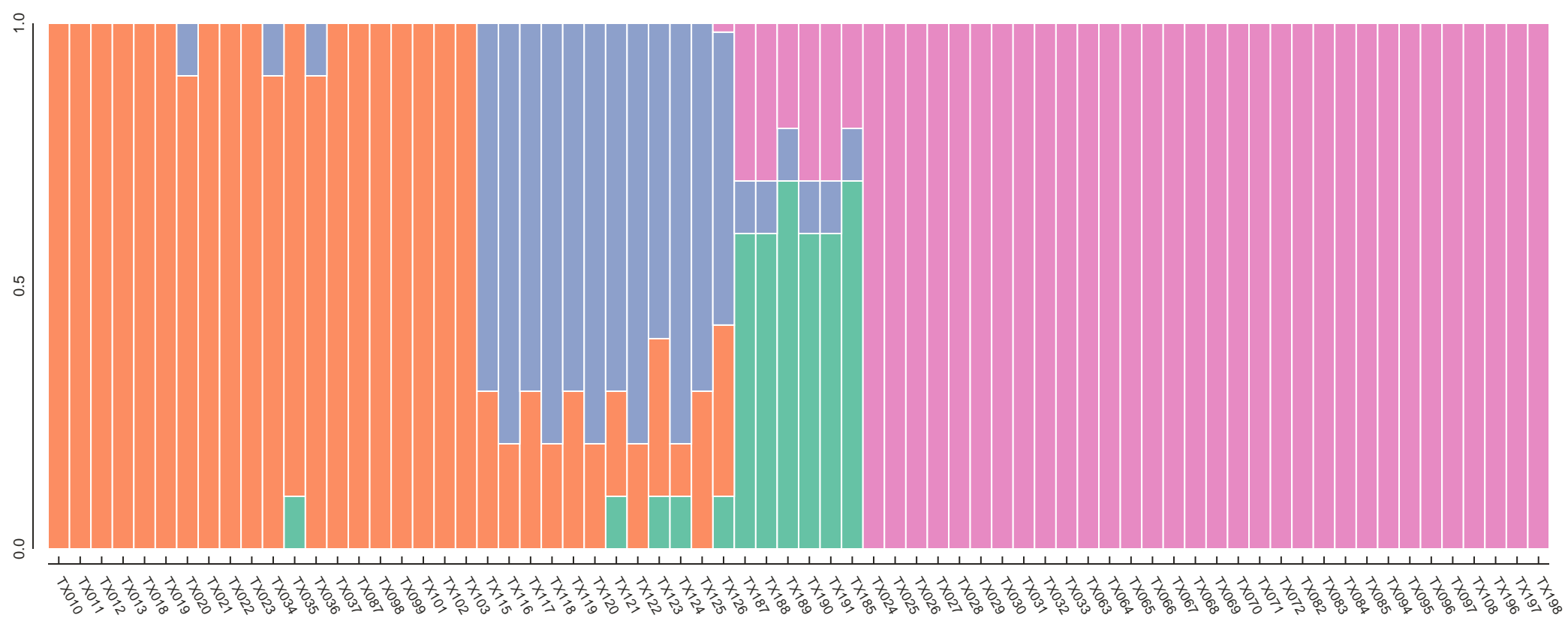

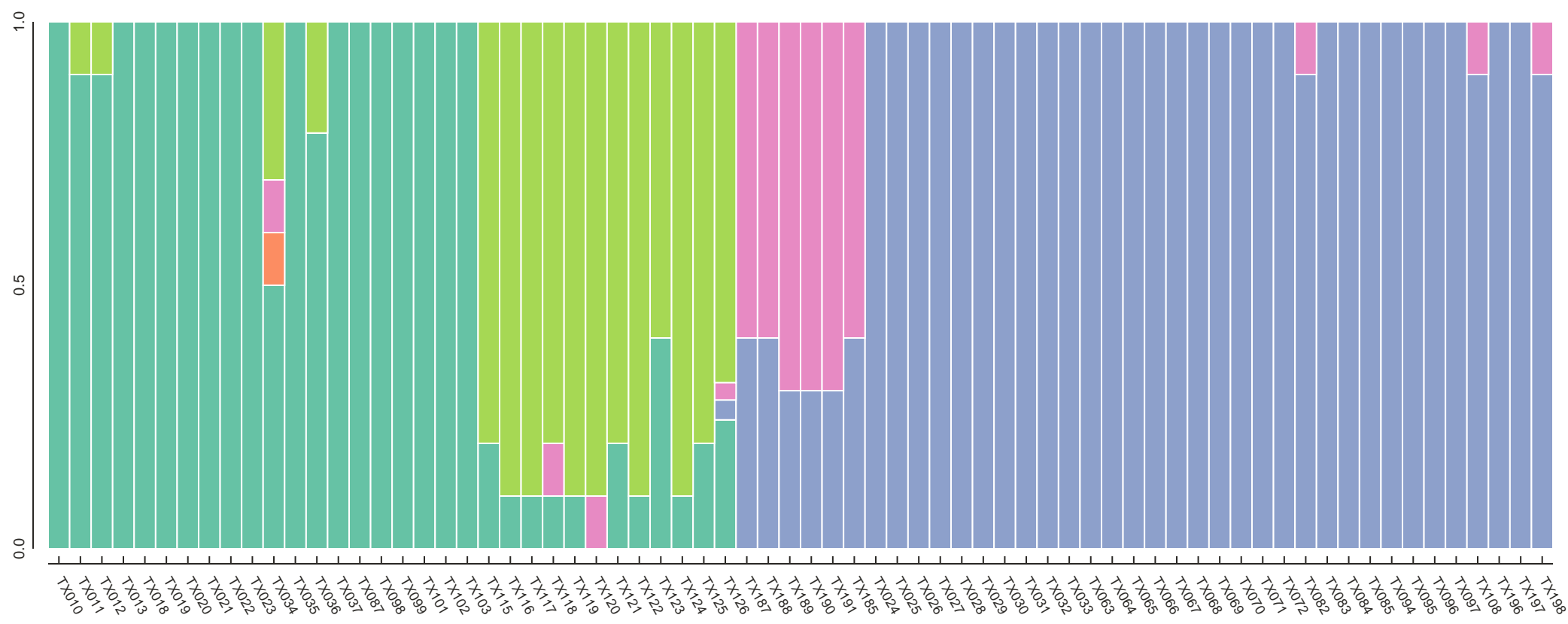

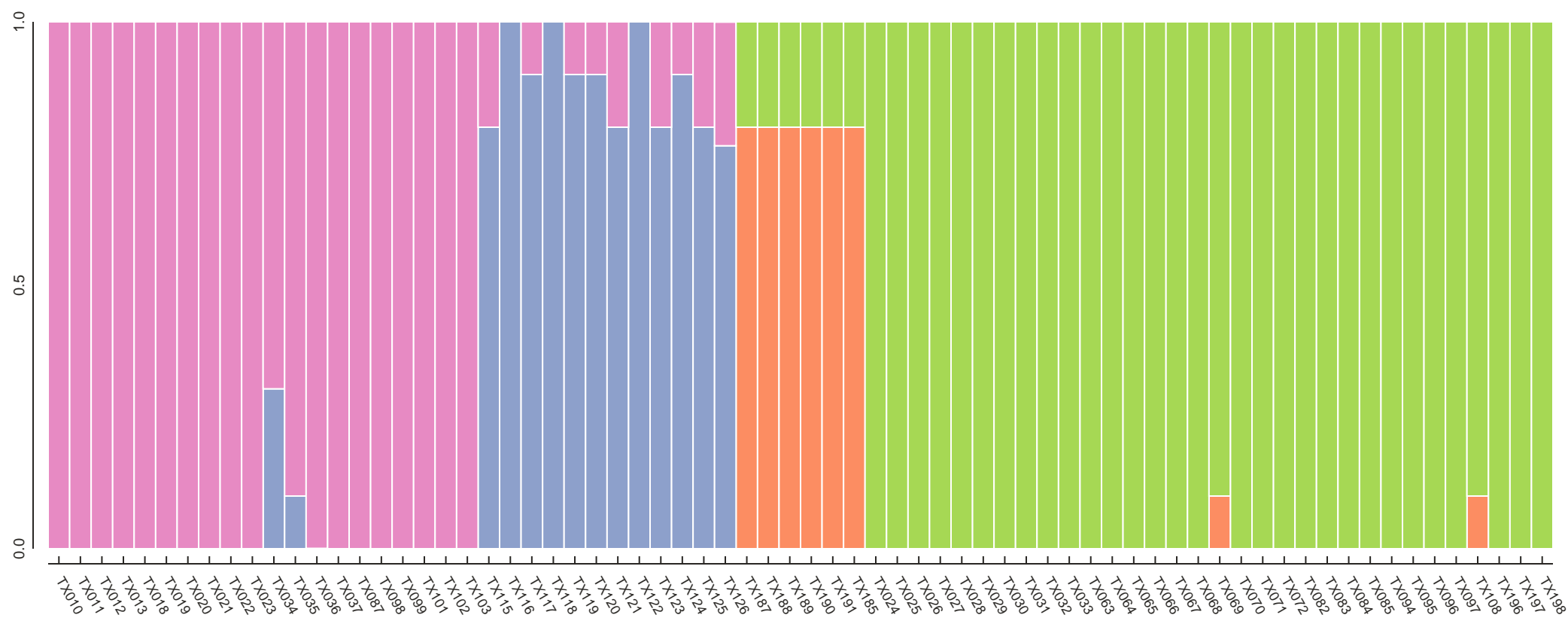

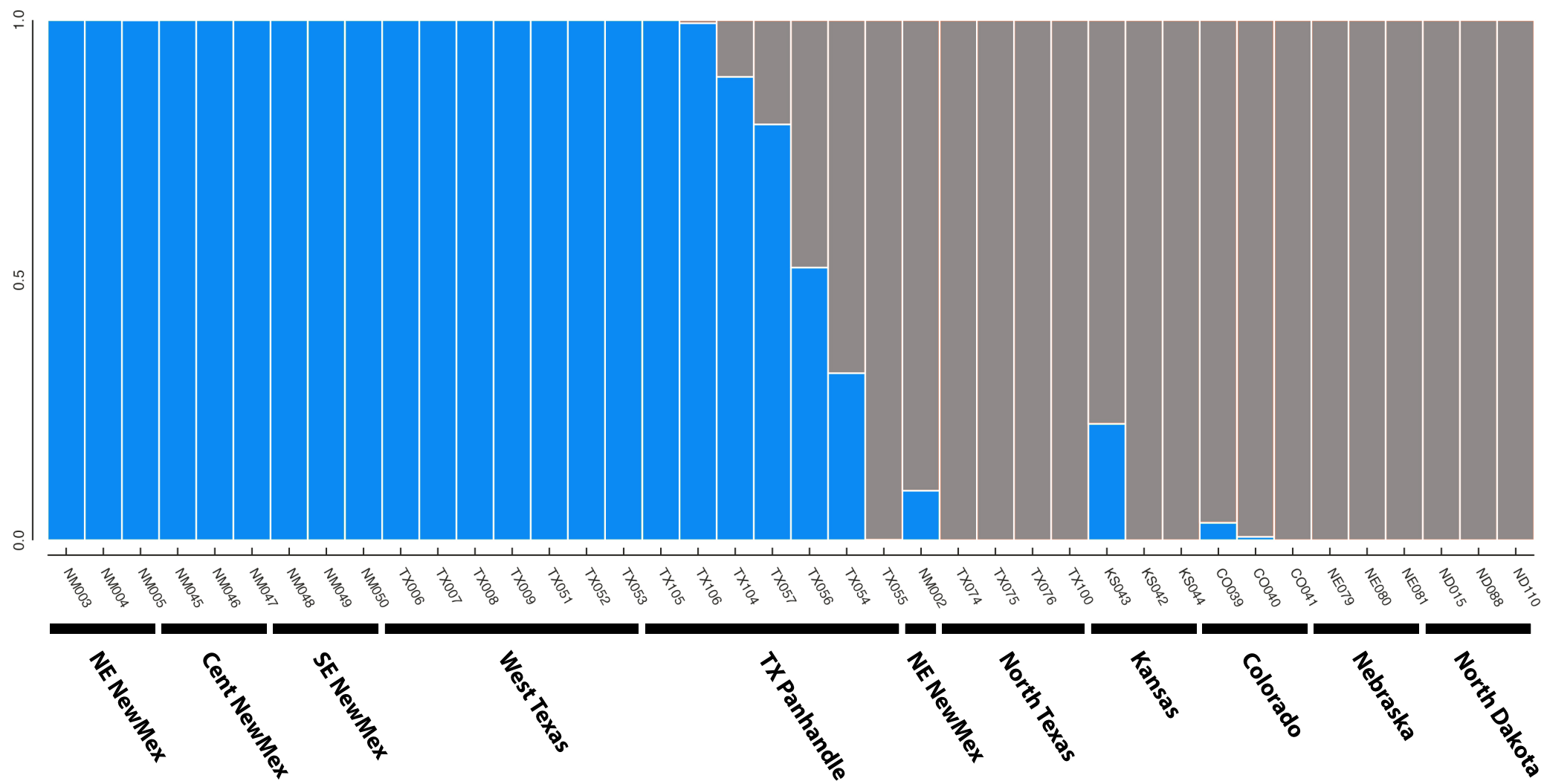

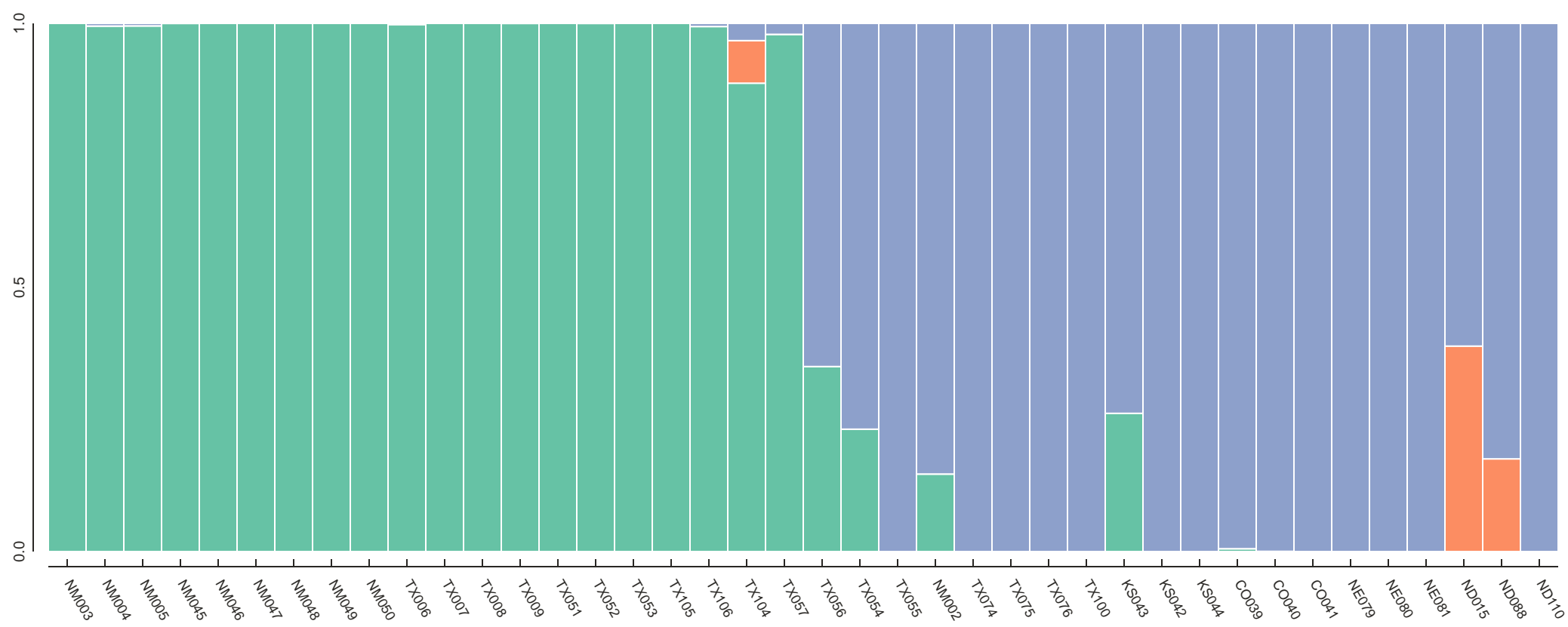

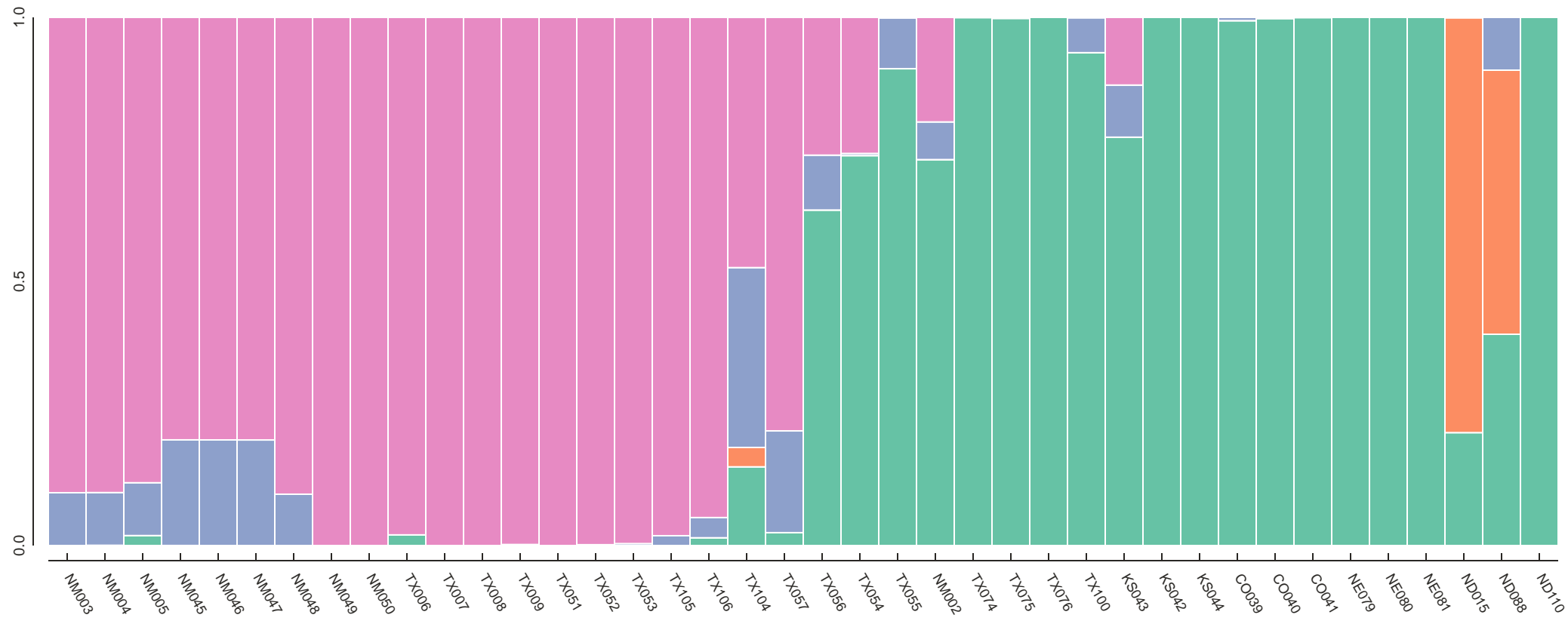

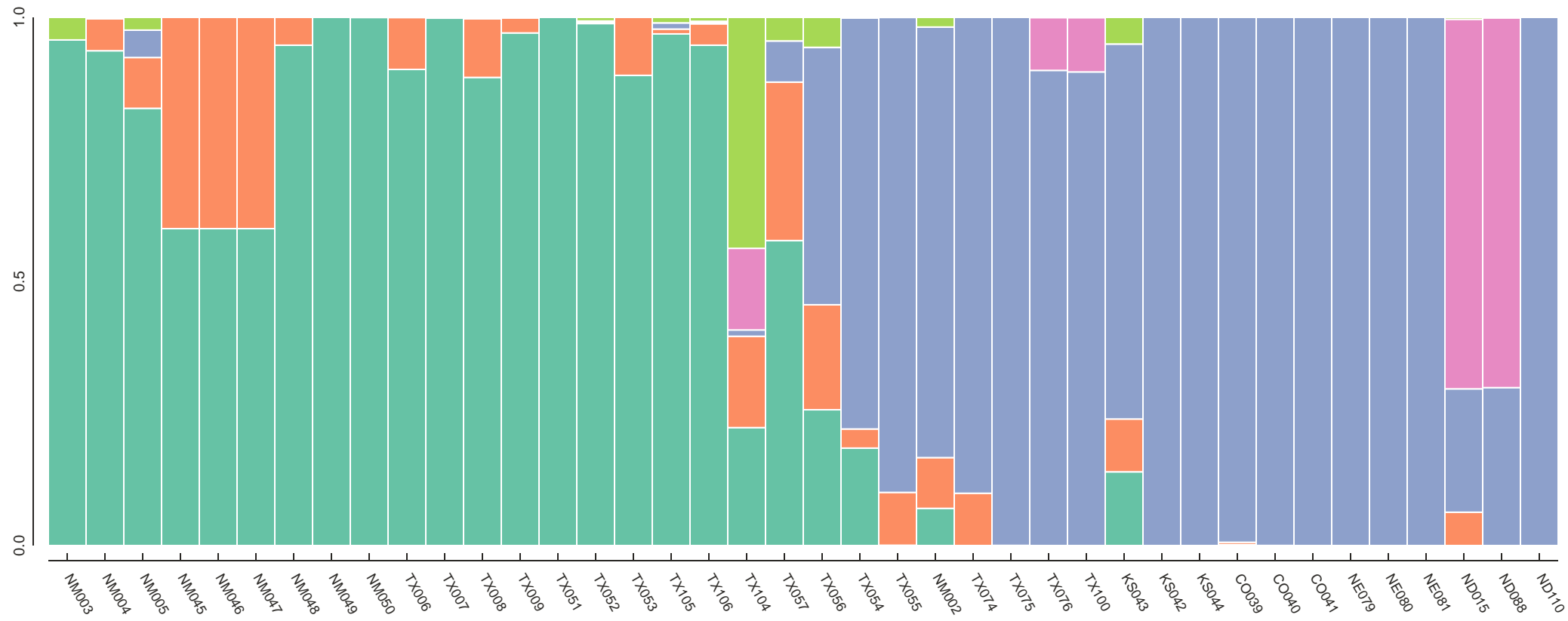

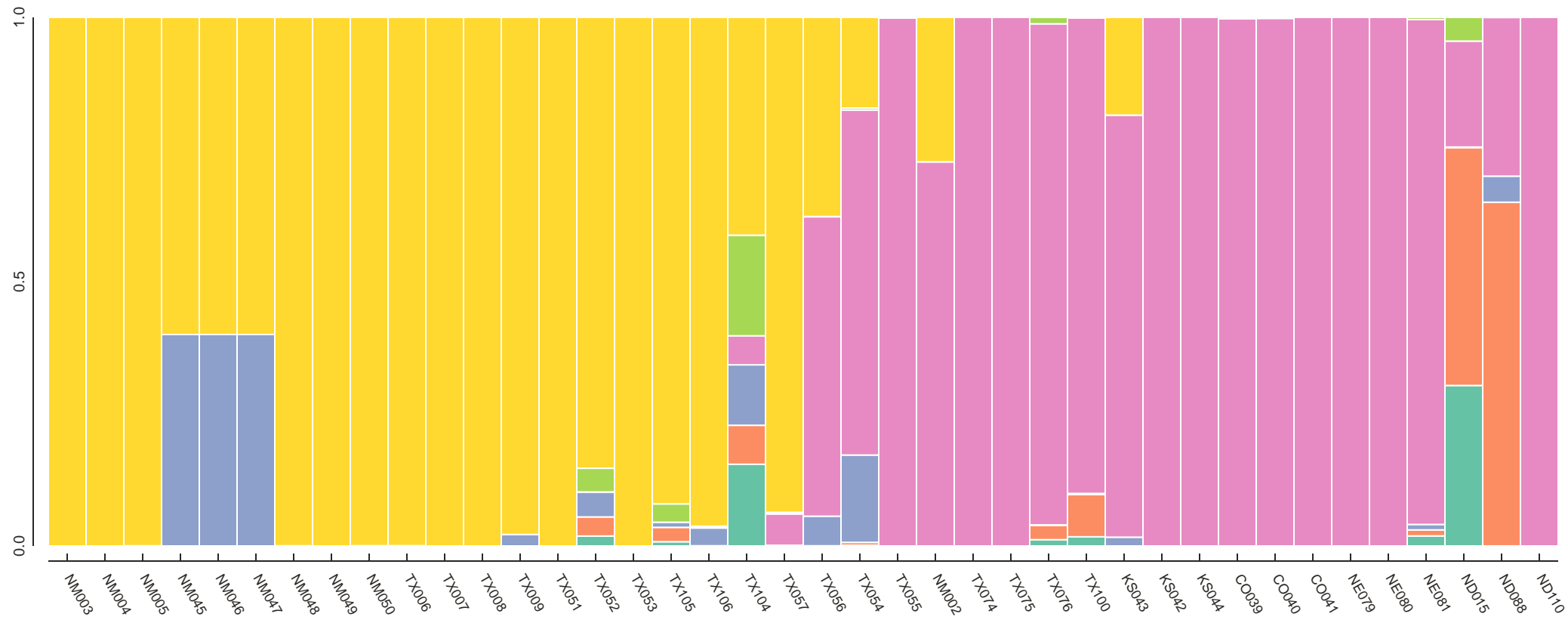

Supplement: Supplementary file 1 — Supplementary Information 1. [file 41598_2024_56875_MOESM1_ESM.pdf]
